# Copy-number dosage regulates telomere maintenance and disease-associated pathways in neuroblastoma

**DOI:** 10.1016/j.isci.2024.110918

**Published:** 2024-09-10

**Authors:** Martin Burkert, Eric Blanc, Nina Thiessen, Christiane Weber, Joern Toedling, Remo Monti, Victoria M. Dombrowe, Maria Stella de Biase, Tom L. Kaufmann, Kerstin Haase, Sebastian M. Waszak, Angelika Eggert, Dieter Beule, Johannes H. Schulte, Uwe Ohler, Roland F. Schwarz

**Affiliations:** 1 Department of Biology, Humboldt University, Berlin, Germany; 2 Berlin Institute for Medical Systems Biology, Max-Delbrück Center for Molecular Medicine in the Helmholtz Association, Berlin, Germany; 3 Core Unit Bioinformatics, Berlin Institute of Health at Charité–Universitätsmedizin Berlin, corporate member of Freie Universität Berlin and Humboldt-Universität zu Berlin, Charitéplatz 1, Berlin, Germany; 4 Department of Pediatric Oncology/Hematology, Charité-Universitätsmedizin Berlin, Berlin, Germany; 5 BIFOLD - Berlin Institute for the Foundations of Learning and Data, Berlin, Germany; 6 Department of Electrical Engineering & Computer Science, Technische Universität Berlin, Marchstr. 23, 10587 Berlin, Germany; 7 Centre for Molecular Medicine Norway (NCMM), Nordic EMBL Partnership, University of Oslo and Oslo University Hospital, Oslo, Norway; 8 Department of Pediatric Research, Division of Pediatric and Adolescent Medicine, Rikshospitalet, Oslo University Hospital, Oslo, Norway; 9 Institute for Computational Cancer Biology (ICCB), Center for Integrated Oncology (CIO), Cancer Research Center Cologne Essen (CCCE), Faculty of Medicine and University Hospital Cologne, University of Cologne, Cologne, Germany; 10 Department of Neurology, University of California, San Francisco, San Francisco, CA, USA; 11 German Cancer Consortium (DKTK), partner site Berlin, and German Cancer Research Center (DKFZ), 69120 Heidelberg, Germany

**Keywords:** Genomics, Molecular biology, Cell biology, Transcriptomics

## Abstract

Telomere maintenance in neuroblastoma is linked to poor outcome and caused by either telomerase reverse transcriptase (TERT) activation or through alternative lengthening of telomeres (ALT). In contrast to TERT activation, commonly caused by genomic rearrangements or MYCN amplification, ALT is less well understood. Alterations at the ATRX locus are key drivers of ALT but only present in ∼50% of ALT tumors. To identify potential new pathways to telomere maintenance, we investigate allele-specific gene dosage effects from whole genomes and transcriptomes in 115 primary neuroblastomas. We show that copy-number dosage deregulates telomere maintenance, genomic stability, and neuronal pathways and identify upregulation of variants of histone H3 and H2A as a potential alternative pathway to ALT. We investigate the interplay between *TERT* activation, overexpression and copy-number dosage and reveal loss of imprinting at the *RTL1* gene associated with poor clinical outcome. These results highlight the importance of gene dosage in key oncogenic mechanisms in neuroblastoma.

## Highlights

• Systematic evaluation of allele-specific gene dosage effects in neuroblastoma
• Dosage is linked to telomere maintenance, genomics stability, and neuronal pathways
• Histone variants of histone H3 and H2A might be involved in telomere maintenance
• Loss-of-imprinting of RTL1 possibly related to poor prognosis in neuroblastoma

## Graphical abstract

**Figure:**
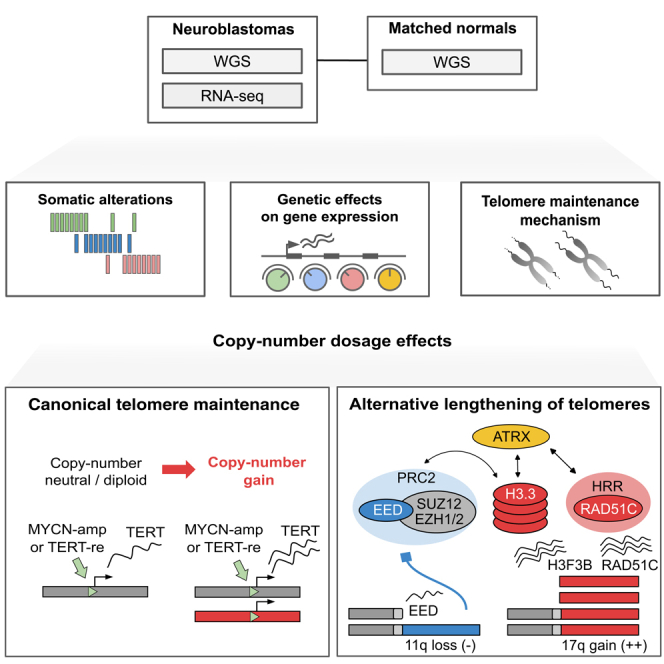

## Introduction

Neuroblastoma is the most common extracranial solid tumor in children accounting for 6–10% of malignancies^1^ and 9% of pediatric cancer deaths.^2^ Clinical manifestations range from high-risk cases with poor survival rates despite multimodal treatment to tumors that spontaneously regress without intervention.^3^ Incidence is highest in the first year of life and only 5% of diagnoses are made in patients older than ten years.^1^ Survival rates rapidly decrease for diagnosis made in children older than 1 year of age.^2^

Genetically, neuroblastoma is characterized by a low single-nucleotide variant (SNV) burden and only few recurrently mutated genes,^4^ but frequent somatic copy-number alterations (SCNAs).^5^^,^^6^^,^^7^ Amplification of the oncogenic transcription factor *MYCN*, often through extrachromosomal circular DNAs (ecDNA),^8^^,^^9^ is found in 20% of tumors and a key clinical indicator for high-risk disease and poor prognosis.^3^^,^^10^ In addition, recurrent segmental gains and losses, including 17q gains and losses of 1p and 11q^6^^,^^11^^,^^12^ are associated with unfavorable outcomes.^13^ Conversely, numerical alterations in chromosomes and whole-genome doubling (WGD) are features associated with better survival rates.^3^ In line with adult tumors,^14^ amplifications of e.g., *MYCN* and *ALK* and their downstream targets^15^^,^^16^ and larger segmental gains and losses correlate well with local RNA levels,^17^^,^^18^ which in turn predict patient survival.^15^^,^^16^^,^^18^

Telomere maintenance leading to replicative immortality^19^ is a common mechanism in high-risk neuroblastoma,^20^^,^^21^^,^^22^ while ineffective telomere maintenance may explain the spontaneous regressions observed in low-risk cases, particularly in stage 4S neuroblastomas.^3^^,^^23^ Canonical telomere maintenance (CTM) involves activation of the telomerase reverse transcriptase (*TERT*) gene either indirectly as a downstream effect of *MYCN* amplification, or directly through genomic rearrangements at the *TERT* locus.^20^^,^^22^ Alternative lengthening of telomeres (ALT) in tumors that lack *TERT* activation^24^ involves DNA recombination induced by breaks at telomeric sequences^25^ and is characterized by single stranded telomeric (CCCTAA)_n_ sequences.^26^ Generally, ALT is associated with loss of function mutations in the *ATRX* and *DAXX* genes^27^ as well as missense mutations in *H3F3A*^28^ and has been found in 50% of all cancer types of the Pan-Cancer Analysis of Whole Genomes (PCAWG) cohort.^29^ Affected tumors show excess telomere length compared to normal tissue and other tumors, including those with activated *TERT*.^29^ In neuroblastoma ALT is associated with ATRX alterations,^20^^,^^30^^,^^31^ significantly enriched in relapse cases and associated with poor outcome independent of other risk markers.^21^^,^^31^^,^^32^ While previous studies have highlighted the molecular characteristics of telomere maintenance in neuroblastoma,^20^^,^^30^^,^^31^^,^^33^^,^^34^ *ATRX* mutations were only found in 25% of high-risk and 50–60% of ALT-positive neuroblastomas,^30^^,^^31^^,^^35^ suggesting additional yet unrecognized mechanisms of ALT activation. Telomere maintenance is therefore a key phenotypic property of neuroblastoma cells and a prime example of phenotypic convergence in cancer evolution,^36^ where multiple somatic aberrations act individually or in concert to activate telomere maintenance pathways by modulating gene expression.

To reveal such mechanisms, we here investigate the effect of genomic instability on total and allele-specific gene expression (ASE) and telomere maintenance in 115 primary neuroblastomas. We analyze whole genome sequencing (WGS) and RNA-seq from tumors and WGS of matched normals, characterize local genetic effects on gene expression variability, and examine the role of copy-number dosage in telomere maintenance and survival.

## Results

### Cohort overview

**Figure 1:**
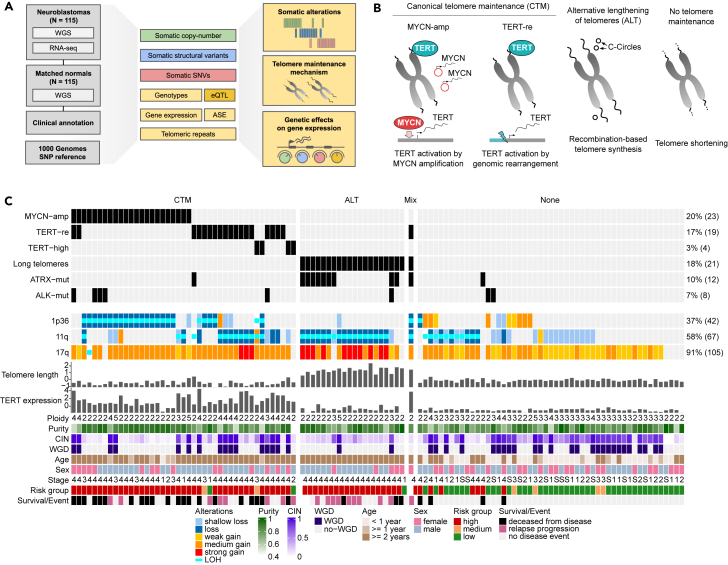
Molecular profiles of 115 neuroblastoma tumors (A) Overview of integrated data processing of tumor WGS and RNA-seq as well as WGS of matched normal samples from 115 neuroblastomas to determine somatic alterations, telomere maintenance mechanisms and genetic effects on gene expression. (B) Overview of telomere maintenance mechanisms: canonical telomere maintenance is characterized by *TERT* activation. In neuroblastoma *TERT* activation is frequently induced by *MYCN* amplification or genomic-rearrangements at the *TERT* locus. ALT is characterized by excess telomere length and C-circles. (C) Molecular and clinical characterization of 115 neuroblastoma primary tumors (columns). ALT, Alternative lengthening of telomeres; *MYCN*-amp, *MYCN* amplification; *TERT*-re, *TERT* rearrangement; *ATRX*-mut, *ATRX* mutation; *ALK*-mut, *ALK* mutation; LOH, Loss of heterozygosity; WGD, Whole-genome doubling; CIN, Chromosome instability index; TMM = None indicates no evidence for a telomere maintenance mechanism detected.

We assembled a cohort of matched tumor WGS and RNA-seq and normal WGS from blood from 115 primary neuroblastoma samples, including 52 samples from the University Hospital of Cologne, previously reported in the study by Peifer et al.,^20^ and 63 new specimens from the GPOH-NB2004 clinical trial. All samples were jointly processed using unified pipelines to limit cohort-specific biases (Figure 1A; STAR methods) and stratified according to the GPOH-NB2004 clinical trial protocol^37^ into 66 high-risk, 6 medium-risk, and 43 low-risk tumors (Figure S1) and equipped with clinical annotations including age, sex, and survival times (Table S1).

Normal samples from blood were genotyped and phased at common germline variant sites (STAR methods). Total and ASE was quantified using phased variants and variant effects on gene expression in *cis* were quantified by genome-wide expression quantitative trait locus (eQTL) mapping (STAR methods).^14^ To explore the mutational landscape we determined somatic SNVs, structural variants (SVs), and allele-specific SCNAs from WGS (STAR methods).

### Telomere maintenance status of 115 primary neuroblastomas

We first set out to determine the primary telomere maintenance mechanism (Figure 1B) and genetic alterations across all 115 tumors by examining somatic SNV, SV, SCNA, and expression data as well as WGS-based estimates of telomere length (STAR methods). We found *MYCN* amplifications in 23 tumors (20%), rearrangements affecting the *TERT* locus in 19 tumors (17%) and *ATRX* mutations in 12 tumors (10%), comprising 7 focal deletions, 4 missense or nonsense mutations and one tumor affected by a structural rearrangement (NBL54) (Figures 1C and S2).

To determine the ALT status of tumors we estimated telomere lengths relative to the matched normal tissue by the abundance of telomeric repeat sequences from WGS (Figure S3A; STAR methods).^38^ We found 21 tumors to show increased telomere lengths, of which we assigned 20 to the ALT group, as one (NBL54) also harbored a *TERT* rearrangement and upregulation of *TERT* (Figures 1C and S3A). We validated our ALT classification by comparison against experimentally determined status of ALT-associated PML-nuclear bodies (APB)^21^ and the presence of circular partially single stranded extrachromosomal telomeric repeat sequences (C-circles)^31^ in subsets of 52 and 36 of analyzed tumor samples, respectively (Figures S3B and S3C). We found strong correspondence between our ALT classification and APBs (*p* = 5.47 × 10^−9^, one-sided Fisher’s exact test; sensitivity: 0.86; specificity: 0.97) as well as C-circles (*p* = 4.33 × 10^−8^, one-sided Fisher’s exact test; sensitivity: 0.91; specificity: 1.00). ALT was detected in 19 of 66 (29%) high-risk tumors, similar to the ALT prevalence estimate in an independent neuroblastoma cohort.^35^ Among ALT tumors 9/20 (45%) harbored ATRX alterations and a single tumor (NBL49) a *H3F3A* missense mutation (p.A48E). We did not find any *DAXX* alterations in ALT tumors of our cohort. While *ATRX* altered samples had significantly longer telomeres (*p* = 1.72 × 10^−6^, one sided Wilcoxon rank-sum test) (Figure S4), 10 out of 20 ALT samples (50%) did not show any mutation in previously described ALT-associated genes, pointing toward alternative activation of the ALT pathway. We found 8 tumors without *MYCN* amplification or *TERT* rearrangements to show high *TERT* expression (Figure S5; STAR methods), of which 4 were not classified as ALT and assigned to the TERT-high group (STAR methods).

Except for three tumors, *MYCN* amplifications, *TERT* rearrangements, and long telomeres were mutually exclusive (Figure 1C), in support of convergence toward a common high-risk phenotype characterized by telomere maintenance.^20^^,^^21^^,^^22^ MYCN amplifications were also mutually exclusive to ATRX alterations, corroborating findings on incompatibility of these two molecular traits.^39^ Of 43 low risk tumors 40 (93%) showed neither increased telomere length (log ratio >0.5) nor elevated *TERT* expression (*Z* score > −0.10). Interestingly, active telomere maintenance was predicted in three low risk tumors (NBL09, NBL23, and CB2035), which all showed disease progression. Notably, we did not find any *MYCN* amplifications in ALT samples and only a single sample with both *TERT* rearrangement and long telomeres (NBL54).

In summary, 43 tumors were classified as showing CTM characterized by *TERT* activation through *MYCN* amplification, *TERT* rearrangement or high *TERT* expression. 20 tumors showed *ALT*, one tumor exhibited a *mixed phenotype* (CTM and ALT), and in 51 tumors no evidence for any telomere maintenance mechanism was found (Figures 1B and 1C; STAR methods). 58 of 66 high-risk tumors (88%) were classified as CTM or ALT in contrast to 7 high-risk tumors (11%) without signs of telomere maintenance.

### Quantifying genomic instability

**Figure 2:**
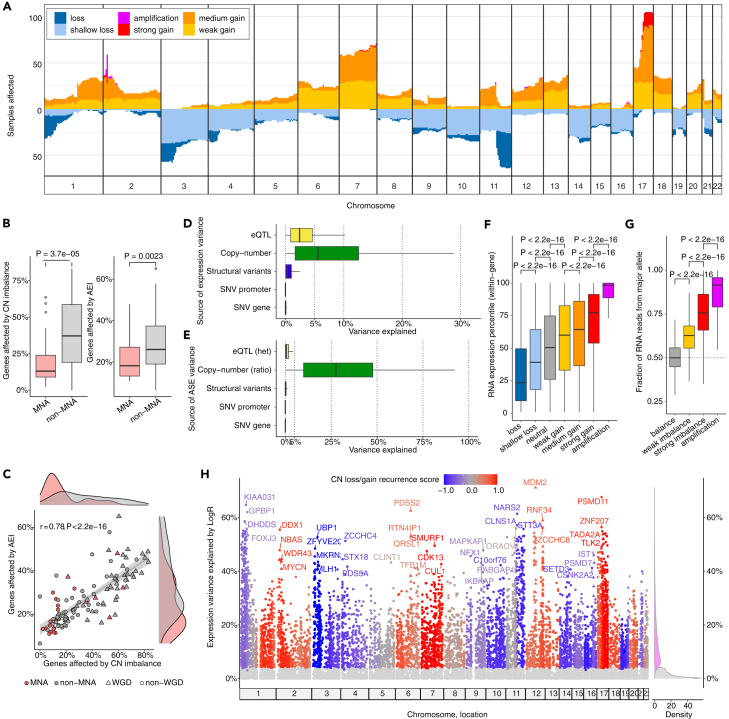
Genetic- and allelic dosage effects in gene regulation (A) Number of samples affected and copy-number state summarized in 5 Mb genomic bins on chromosomes 1–22. (B) Number of genes affected by copy-number imbalance and AEI for samples with (*n* = 23) and without (*n* = 92) *MYCN*-amplification (MNA). (C) Number of genes affected by copy-number imbalance and allelic expression imbalance (AEI) per sample. Gray line represents linear regression fit, with light gray ribbon showing the 95% confidence interval. Pearson correlation coefficient *r* and *p*-value *P* are shown. CN, copy-number. WGD, whole-genome doubling. (D) Quantification of genetic effects on the variance of total expression of expressed genes (*n* = 13,632). (E) Quantification of genetic effects on the variance of allele-specific expression (ASE) of ASE informative genes (*n* = 10,656). (F) Distribution of within-gene expression percentile per sample and gene by copy-number state: loss (*n* = 66,030), shallow loss (*n* = 233,696), neutral (*n* = 1,322,030), weak gain (*n* = 160,861), medium gain (*n* = 177,750), strong gain (*n* = 8,453), amplification (*n* = 383). (G) Proportion of RNA reads from major allele per sample and gene by copy-number balance state: balance (*n* = 1,224,053), weak imbalance (*n* = 549,177), strong imbalance (*n* = 165,790), amplification (*n* = 383). (H) Genome-wide gene-level copy-number dosage effects. Significant copy-number dosage effect genes (FDR <0.05, Benjamini-Hochberg) indicated in color scale, others in light gray. Boxplot midlines in (B, D, E, F, and G) mark median; upper and lower hinges extend to first and third quartile; upper and lower whiskers extend to the smallest and largest value max. 1.5 × IQR; *p* value of two-sided Wilcoxon test is shown between groups in (B, F, and G).

We next determined allele-specific SCNAs and overall ploidy from WGS (STAR methods) and classified copy-number segments into states *loss*, *shallow loss*, *neutral*, *weak gain*, *medium gain*, *strong gain*, and *focal amplification* (Figure 2A) and into allelic imbalance states *balance*, *weak imbalance*, *strong imbalance*, *amplification*, and *LOH* (Figure S6; STAR methods). We further detected WGD events by phylogenetic copy number analysis as recently described.^40^ On average 50% of the genomic regions harbored SCNAs, 31% of genomic regions showed gains and losses relative to ploidy, and 44 tumors (38%) showed WGD (Figures 1C and S7). We identified gains in 17%, losses in 15%, and amplifications in <0.1% of genomic regions, with distinct hotspots on the cohort level (Figure 2A). We found WGDs to be overrepresented in tumors without telomere maintenance (26 of 51, expected 20, *p* = 0.03, Fisher-exact test), as opposed to ALT (2 of 20, expected 8, *p* = 0.01). Tumors with CTM in contrast did now show enrichment (16 of 43, expected 16, *p* = 1.0).

Next, we determined ASE in all 115 tumors. Briefly, read counts from RNA-seq were tallied up at heterozygous germline variants and aggregated to haplotype counts per gene using statistical phasing (STAR methods). In line with prior observations,^41^ we found lower genomic instability in MYCN-amplified tumors compared to non-amplified tumors, visible both in the number of copy-number-imbalanced genes (*p* = 3.7 × 10^5^) and genes with ASE (*p* = 0.0023) (one-sided Wilcox rank-sum test) (Figure 2B). Interestingly, we also identified 4 out of 23 (17%) *MYCN* amplified tumors with significantly more allelically imbalanced genes compared to non-*MYCN*-amplified samples (37% of genes). All 4 tumors showed signs of WGD and overall high chromosomal instability (>80%) (Figure 2C; Table S1) and 3 out of 4 of these patients died from the disease. Increased genomic instability in *MYCN* amplified tumors thus might cf. an additional risk factor similar to earlier findings on chromosomal losses in *MYCN* amplified tumors.^12^

Focal amplifications were detected in 32 tumors and recurrently affected 35 genes, including COSMIC census genes^42^ *MYCN*, *ALK*, *CDK4*, *LRIG3*, *MDM2*, and *PTPRB* (Figure S8). Genes amplified in three or more tumors were exclusively detected in *MYCN* co-amplified regions on 2p24, likely associated with ecDNA presence. LOH affected 5% of genomic regions, and 1p36 LOH was found in 26 tumors (22%) of which 18 also showed amplification of *MYCN*. Shallow losses of 1p36 without LOH were detected in 6 tumors (5%). LOH of 11q was found in 42 tumors (37%), out of which 17 affected ALT tumors, 8 affected tumors with *TERT* rearrangements and 3 *MYCN*-amplified tumors. We found 11q losses without LOH in 23 tumors (20%), of which 19 were classified as shallow losses. 11q loss was found in 18 of 19 ALT tumors (90%), in line with previous reports on frequent 11q losses in ALT.^31^ Gains of 17q were highly abundant and affected 104 tumors (90%). Interestingly, 10 out of 13 strong 17q gains (78%) affected ALT tumors, suggesting that relatively higher 17q copy-numbers may be linked to the ALT phenotype in neuroblastoma. SV analysis indicated substantial heterogeneity in SV burden between tumors (Figure S9A) and revealed frequent interchromosomal rearrangements (Figures S9B and S9E). We analyzed the frequency of somatic SVs in 500 kb segments along the genome and detected recurrent SV breakpoints at the *MYCN*, *TERT*, *ATRX*, *11q13*, and *17q21* loci (Figures S9C–S9E), confirming previous findings on SV frequencies in a subset of tumors analyzed.^20^

To investigate the effect of SCNAs on patient survival systematically we associated allelic copy-number imbalances on the level of chromosome arms and in non-overlapping 5Mb bins with mortality (STAR Methods) and found expected associations at 1p and the *MYCN* locus as well as a yet undescribed association of 17p imbalance (Figures S10A–S10C). Five tumors of deceased patients harbored extreme copy-number imbalances (>0.9) due to loss of 17p (Figure S11A), pointing toward elevated risk conferred through chromosomal loss. However, also 10 out of 26 donors (38%) with tumors harboring imbalanced gains died from the disease. We compared survival probabilities using the Kaplan-Meier method and found that survival was significantly reduced for tumors with 17p imbalance (*p* = 5.2 × 10^−4^) (Figure S11B). Similarly, Cox proportional hazard regression showed that 17p imbalance is significantly associated with mortality (*p* = 1.44 × 10^−5^), independent of *MYCN* amplification (*p* = 4.32 × 10^−6^) (Figure S12). Notably, 17p LOH is frequent in neuroblastoma cell lines,^43^ but its occurrence in primary neuroblastoma is less well described. Interestingly, we did not find *TP53* missense mutations or SVs, suggesting that 17p loss might act through downregulation of neuronal genes (Figures S11C and S11D; Table S2) or through a second hit in *TP53* that occurred after the sampling time point.

### Copy-number dosage specifically regulates cancer-related pathways in neuroblastoma

We next sought to identify the effects of genetic alterations and to quantify their contribution to gene regulation in neuroblastoma. We used linear models to predict both total gene expression and the ASE ratio per gene from its lead *cis*-eQTL variant, proximal SV breakpoints, copy-number status, and local mutational SNV burdens in promoter and gene body (see STAR methods and^14^). For ASE analysis, an average of 5,768 (2,691–7,544) expressed genes were considered per tumor. In keeping with the literature, we found SCNAs to have the strongest effect among all genetic factors on both ASE and total gene expression,^44^ explaining an estimated 30.3% and 8.0% of variance in ASE and total expression, respectively (Figures 2D and 2E), and demonstrating a clear allele-specific copy-number dosage effect on gene expression on average (Figures 2F and 2G). Lead germline *cis*-eQTL variants were the second largest genetic contributor explaining 1.6% of variance in ASE and 2.6% of variance in total gene expression. Despite emerging evidence of targeted *cis*-deregulation in neuroblastoma,^20^^,^^33^^,^^45^ overall somatic SVs and SNVs explain the least amount of variance in ASE and total expression with less than 1.0% and 1.2%, respectively, on average, in line with recent findings in adult tumors.^14^

Even though SCNAs exhibit a strong allelic dosage effect on gene expression, transcription levels of genes are subject to transcriptional adaptations and buffering.^46^^,^^47^ To investigate dosage sensitivity in our cohort systematically, we examined copy-number components in our linear models and found statistically significant copy-number effects that explain between 2.4% and 71.0% of observed variance in gene expression (Figures 2H and S13). We ranked all protein-coding genes by expression variance explained and tested for pathway enrichment using gene set enrichment analysis (GSEA, STAR methods). We found 69 Reactome pathways enriched (FDR <0.05) for copy-number dosage effects (Table S3), of which 25 remained after accounting for overlapping gene sets (Figure S14). Notably, dosage sensitive genes were enriched in pathways involved in cell cycle and DNA repair, and in regulation of tumor suppressor genes *TP53*, *PTEN*, and *RUNX3*. In contrast, conducting the same GSEA analysis on genes ranked by total copy-number alone did not yield any significant pathway enrichment.

### Copy-number dosage modulates TERT expression in telomere-maintaining tumors

**Figure 3:**
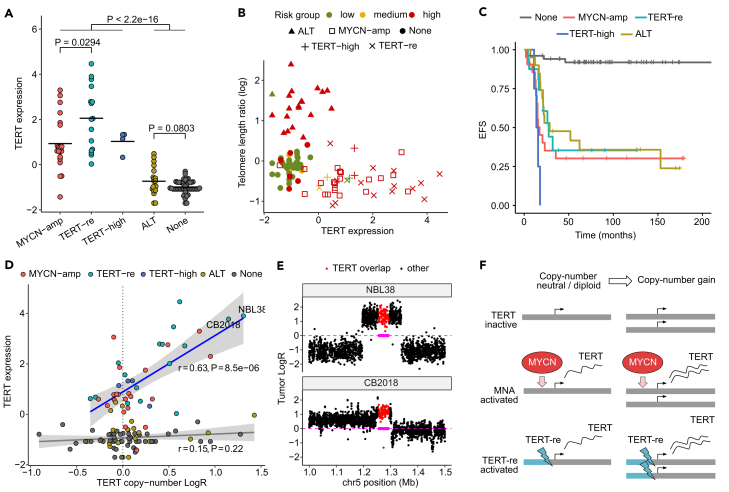
Copy-number dosage effects in canonical telomere maintenance (A) Comparison of *TERT* expression between tumors by molecular telomere maintenance characteristics. Horizontal bars indicate group mean. Two-sided Wilcoxon test *p* value is shown between groups. (B) Telomere length and *TERT* expression per sample. (C) Kaplan-Meier estimate of event free survival (EFS) by telomere maintenance mechanism in primary tumors. (D) Copy-number LogR and expression of *TERT* per sample. Linear regression line of samples with canonical telomere maintenance (*MYCN*-amp, *TERT*-re, *TERT*-high) in blue, regression line of other samples in gray. Gray ribbons indicate 95% confidence intervals. Pearson correlation coefficient *r* and *p*-value *P* are shown. (E) Local *TERT* copy-number gains in tumors NBL38 and CB2018 labeled in (D). Baseline (tumor LogR = 0) and *TERT* gene boundaries as magenta dashed line and box, respectively. (F) Copy-number gains induce higher *TERT* expression in tumors of activated *TERT*. Tumors with more than one molecular characteristic not shown in (A, B, C, and D). *MYCN*-amp, *MYCN* amplification; *TERT*-re, *TERT* rearrangement; *TERT*-high, high *TERT* expression; ALT: Alternative lengthening of telomeres.

We queried *TERT* gene expression in all tumors and found both *MYCN* amplified and *TERT*-rearranged samples to have significantly higher *TERT* expression than those lacking both molecular features (Figure 3A), in line with previous observations.^20^^,^^48^ Comparison of *TERT* expression with telomere length estimates confirmed the existence of two distinct groups of high risk tumors: those with high *TERT* expression but short telomeres and those with low *TERT* expression but increased telomere length, indicative of ALT (Figure 3B). Event-free survival was significantly reduced for all of the three inferred telomere maintenance mechanisms compared to tumors without telomere maintenance (*MYCN*-amp: *p* = 1.01 × 10^−6^; *TERT*-re: 5.56 × 10^−5^; *TERT*-high: 1.16 × 10^−7^; ALT: *p* = 2.47 × 10^−5^; Cox proportional hazards regression) (Figure 3C). When considering overall survival, we found significant associations only with MYCN-amp and TERT status, but not with ALT (MYCN-amp: *p* = 1.17 × 10^−5^; TERT-re: *p* = 0.036; TERT-high: 3.16 × 10^−5^; ALT: *p* = 0.241; Cox proportional hazards regression) (Figure S15).

Copy-number dosage analysis of the *TERT* locus revealed that in CTM tumors (*MYCN*-amp, *TERT*-re, *TERT*-high) *TERT* dosage is significantly correlated with *TERT* expression (Figure 3D, Pearson’s r = 0.63, *p* = 8.5 × 10^−6^) as opposed to tumors with ALT or without telomere maintenance (r = 0.15, *p* = 0.22), independent of sample purity (*p* = 0.48, ANOVA F-statistic). *TERT*-re tumors harbored frequent *TERT* copy-number gains with breakpoints proximal to the *TERT* locus (Figure S16). Among three tumors with strongest *TERT* expression, two (NBL38, CB2018) harbored focal *TERT* gains (Figure 3E), indicating targeted upregulation of activated *TERT* by copy-number gains. None of the 3 *TERT*-high tumors showed *TERT* copy-number gains and thus these measures were not significantly correlated in this group either (Pearson’s r = −0.81, *p* = 0.19). Our findings show that SCNAs adjust the regulatory landscape of neuroblastoma toward dysregulation of key cancer pathways and that copy-number gains effectively upregulate *TERT* in tumors with CTM (Figure 3F), with highest telomerase expression found in tumors with both *TERT* activation and copy-number gains.

### 11q loss and 17q polysomy link alternative lengthening of telomeres to upregulation of histone variants

**Figure 4:**
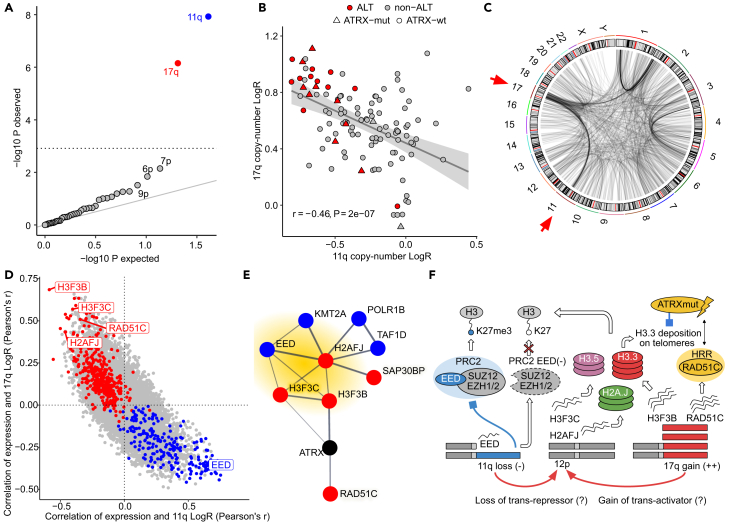
Copy-number alterations and upregulation of histone variant genes in alternative lengthening of telomeres (A) Association *p*-values of ALT and coverage LogR per chromosome arm. Significant observations in red (gain) and blue (loss), others in gray. Significance threshold (FWER 0.05) demarcated by gray dotted line. (B) 11q and 17q LogR per sample indicating ALT and *ATRX* status. Gray line represents linear regression fit, with light gray ribbon showing 95% confidence interval. Pearson correlation coefficient *r* and *p*-value *P* are shown. (C) Genome-wide somatic structural variant breakpoints. Breakpoints of frequent rearrangements between chromosome arms 11q and 17q highlighted by red arrows. (D) Correlation of gene expression and LogR of 17q and 11q. Differentially expressed genes in red (ALT up) and blue (ALT down). (E) STRING database protein interaction network of *ATRX*, *H3F3B*, *H2AFJ* and *H3F3B* and their first order interactions among differentially expressed genes in ALT. Red and blue indicate up- and downregulated genes respectively. *ATRX* is not differentially expressed (black). Histone variant genes and PRC2 complex member *EED* highlighted in yellow. (F) Proposed model of deregulated ATRX-interactions in ALT: 11q and 17q copy-number alterations upregulate histone variants as well as homologous recombination repair (HRR) pathway associated *RAD51C* and impairs PRC2 activity by *EED* downregulation. Reduced PRC2 activity results in H3K27me3 depletion. Mutant *ATRX* (*ATRX* mut.) impairs deposition of upregulated H3.3 and is associated with HRR-dependent elongation of telomeres in ALT.

To investigate if SCNAs are linked to increased telomere length in ALT tumors, we tested each chromosome arm for association between tumor DNA content and ALT using logistic regression, controlling for *ATRX* mutations (STAR methods). We found 11q losses (*p* = 4.83 × 10^−7^, ANOVA Chi-squared test) and 17q gains (*p* = 2.88 × 10^−5^, ANOVA Chi-squared test) to be significantly associated with ALT (Figure 4A), confirming previous observations of frequent 11q loss in ALT^31^ and revealing a yet undescribed association of 17q gain with ALT. We noticed that 11q loss co-occurs with strong 17q gains in 14 tumors and observed an overall negative correlation between DNA content of both chromosome arms across the cohort (*r* = −0.45, *p* = 2.01 × 10^−7^, Pearson’s *correlation*) (Figure 4B), suggesting a genomic rearrangement involving both chromosomes. Indeed, somatic SV analysis revealed 17q to 11q translocations in 19 tumors (Figure 4C), confirming that additional copies of chromosome arm 17q translocate to 11q in the aberrant tumor karyotype.^49^ Notably, 17q gains were identified in 105 of 115 tumors (91%) independent of ALT. However, ALT tumors were significantly enriched in the strongest 17q copy-number gains (Figure S17).

To pinpoint candidate genes contributing to ALT we tested for differential gene expression between ALT and non-ALT tumors, while controlling for *MYCN* amplification status, the presence of *ATRX* mutations and the sex of the patient (STAR methods, FDR 0.05). We found 408 up- and 224 downregulated genes (Figure S18; Table S4) and hypothesized that a subset of these genes might be driven by the ALT-associated SCNAs on 11q and 17q. Correlation between gene expression and DNA dosage of these chromosome arms revealed upregulated histone variant genes *H3F3B* (17q), *H2AFJ* (12p), and *H3F3C* (12p) among genes strongly affected by 17q and 11q dosage (Figure 4D). *H3F3B* (and its paralog *H3F3A*) encode the histone variant H3.3,^50^ which is altered by activating mutations in several pediatric tumor entities, including tumors of the central nervous system^51^^,^^52^ and up to 95% of chondroblastomas.^53^ Interestingly, activating H3.3 mutations triggered ALT in pediatric high-grade glioma regardless of *ATRX* mutation status,^28^ indicating that similarly, H3.3 upregulation may have functional implications in ALT neuroblastomas. *H3F3C*, which encodes for histone variant H3.5 is frequently mutated across different pediatric brain tumors, where alterations were found to be mutually exclusive to those in *TP53* and associated with reduced genome stability.^54^ The *H2AFJ* gene encodes for histone variant H2A.J and is deregulated in melanoma,^55^ breast cancer,^56^ and colorectal cancer, where its upregulation is associated with poor survival.^57^ Taken together, these results suggest that copy-number alterations may deregulate histone variants contributing to epigenetic dysregulation and genome integrity in ALT neuroblastomas.

The genetic effects model (STAR methods) predicted 41% and 60% of expression and ASE variance of *H3F3B* explained by local copy-number effects, indicating that expression of *H3F3B* is directly associated with 17q dosage (Figure S19). However, only 3% of *H2AFJ* and 2% of *H3F3C* expression variance is explained by local copy-number effects on 12p, suggesting upregulation through *trans* effects.

To obtain a quantitative understanding how expression of the identified histone variant genes relates to ALT we predicted presence of ALT from the expression of *H3F3B*, *H3F3C*, and *H2AFJ* using logistic regression. We found expression of *H3F3B* and *H2AFJ*, but not *H3F3C* to be significantly associated with ALT in the presence of the two other genes (*H3F3B*: *p* = 0.001; *H2AFJ*: *p* = 0.008; *H3F3C*: *p* = 0.543; ANOVA), suggesting that expression of *H3F3B* and *H2AFJ* is independently associated with ALT. For an independent validation, we compared the expression levels of *H3F3B* and *H2AFJ* between 130 telomeric c-circle positive and negative neuroblastomas from Hartlieb et al.,^31^ and found significantly higher expression of *H3F3B* (*p* = 3.01 × 10^−4^, ANOVA) and *H2AFJ* (*p* = 0.02, ANOVA) in c-circle positive tumors, confirming their upregulation in ALT (Figure S20).

Despite *ATRX* alterations being significantly associated with longer telomeres, we did not find *ATRX* to be differentially expressed between ALT and non-ALT (Table S4). We speculated that interaction partners of *ATRX* could be subject to deregulation in ALT tumors. To investigate this we obtained direct (first order) predicted protein interactions between *ATRX*, *H3F3B*, *H2AFJ*, *H3F3C*, and other proteins of differentially expressed genes in ALT affected by 11q or 17q dosage (STAR methods). The resulting network predicted high-confidence direct interactions between *ATRX* and differentially expressed histone 3.3 variant gene *H3F3B*, as well as *RAD51C* (Figure 4E). *RAD51C* is essential for homologous recombination repair,^58^ a process utilized in ALT for telomere extension. Notably, gene amplifications and pathogenic variants in RAD51C have been linked to breast and ovarian cancers.^59^^,^^60^ A network module containing *H3F3B*, *H2AFJ*, and *H3F3C* also included deregulated histone methylation factors *EED* and *KMT2A*. *EED* is part of the polycomb repressive complex 2 (*PRC2*), which modulates transcriptional repression by methylation of H3 histones,^61^^,^^62^ and we found *EED* to be downregulated in ALT tumors by 11q-dosage effects (Figures 4F and S21; Table S4). The PRC2 complex is frequently inactivated by *EED* loss in malignant peripheral nerve sheath tumors^63^ and adenosquamous lung tumors.^64^ Upregulation of H3.3 and H3.5 histones and concomitant downregulation of *EED* in ALT point toward a relative depletion of H3K27me3 as a consequence of higher H3 variant histone availability and impaired PRC2 activity (Figure 4F). In a related manner, PRC2 is inhibited by activating H3.3.pK27M mutations in pediatric gliomas^65^^,^^66^^,^^67^ or expression of PRC2 inhibitor *EZHIP* in ependymomas,^68^ both of which we did not find in our cohort (Figure S22).

Recent studies have also demonstrated the binding of telomeric repeat-containing RNAs (TERRAs) to PRC2 and their role in H3K27 tri-methylation of telomeres.^69^ We thus investigated TERRA levels in our cohort (STAR methods) and found ALT tumors to show the highest TERRA expression with substantial variation between tumors (Figure S26). TERRAs were enriched in ALT compared to CTM (*p* = 0.0137, Wilcox rank-sum test) and MYCN-amp tumors (*p* = 0.0186), but not compared to tumors without detectable telomere maintenance (*p* = 0.0532, Wilcox rank-sum test) or TERT-re tumors (*p* = 0.0939, Wilcox rank-sum test) (Figures S26A and S26B). TERRA expression was significantly correlated with relative telomere length (Spearman’s rho: 0.3, *p* = 0.02) across the cohort (Figure S26C).

To validate our findings on an independent dataset we investigated 11q loss and strong 17q gain in the neuroblastoma cohort presented by Gundem et al. 2023,^70^ using ATRX mutations as a surrogate for ALT. Relative losses of 11q (*p* = 0.0029, one-sided Wilcox rank-sum test) and relative gains of 17q were significantly associated with ATRX-mutated samples compared to other tumors (*p* = 0.0124, one-sided Wilcox rank-sum test) (Figure S27), confirming our initial associations of 11q loss and strong 17q gains in ALT in the discovery cohort. We further analyzed dosage effects of key deregulated genes H3F3B (on 17q) and EED (on 11q) in the cohort from Egolf et al. 2019^71^ correlating copy-number with gene expression. We found significant associations of copy-number and expression for H3F3B (*p* = 5.6 × 10–11, Pearson correlation test) and EED (*p* = 3.8 × 10^−7^, Pearson correlation test) (Figure S28), confirming our initial findings of these copy-number dosage effects in the discovery cohort.

Our findings implicate 11q loss and strong 17q gain in ALT neuroblastomas and show that these alterations deregulate *ATRX* interaction partners. They highlight histone variants as key components of ALT-deregulated *ATRX* protein interactions and indicate that activity of the PRC2 complex could be reduced due to attenuated *EED* expression resulting from 11q loss, providing additional evidence for histone-dependent chromatin deregulation by copy-number dosage in ALT neuroblastomas.

### Imprinted RTL1 is upregulated by bi-allelic activation in tumors with unfavorable prognosis

**Figure 5:**
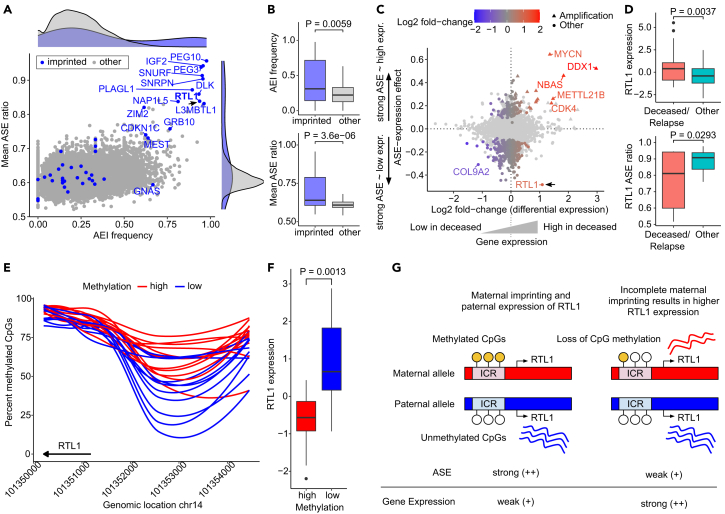
Bi-allelic expression of imprinted gene RTL1 in unfavorable tumors (A) AEI frequency and mean ASE ratio per gene. Imprinted genes in blue, others in gray. (B) AEI frequency (top) and ASE ratio (bottom) by imprinting status per gene: imprinted (*n* = 43), other (*n* = 13,007). (C) ASE-expression effect and differential expression (survival) per gene. Allelic regulated genes in color scale, others in light gray. (D) Gene expression (top) and ASE ratio (bottom) of the *RTL1* gene by survival: Deceased/Relapse (*n* = 46), Other (*n* = 69). (E) Percent methylated CpGs in the genomic region −4 kb to +1 kb relative to *RTL1* gene start. Samples are clustered by above (high) (*n* = 12) and below (low) (*n* = 11) median methylation level in the genomic window shown. (F) *RTL1* expression by methylation clusters shown in (E). (G) Proposed model of *RTL1* upregulation through loss of maternal imprinting. Incomplete imprinting on maternal allele results in bi-allelic expression and upregulation of *RTL1*. ICR: Imprinting control region. Boxplot midlines in (B, D, and F) mark median; upper and lower hinges extend to first and third quartile; upper and lower whiskers extend to the smallest and largest value max. 1.5 × IQR; *p* value of two-sided Wilcoxon test is shown between groups.

Finally, we characterized genes by ASE frequency and average ASE ratio across tumors. The highest ASE ratio (0.96) and frequency (0.98) was found for the *PEG10* gene, which is maternally imprinted in most tissues.^72^ Generally, imprinted genes^73^ including *IGF2*, *DLK1*, *RTL1*, and *L3MBTL1* (Figure 5A) were enriched among the genes with strongest (*p* = 3.6 × 10^−6^) and most frequent ASE (*p* = 0.0059, both Wilcoxon rank-sum test) (Figure 5B), showing that expression imbalance recapitulates imprinting in neuroblastoma.

Since ASE can be caused by either up- or downregulation of gene expression on one parental haplotype, we systematically explored effect directionality by testing for association between ASE and total expression. 10,862 genes that were informative for ASE in at least 20 samples were considered, out of which 455 showed a significant (FDR <0.05) effect of ASE on total gene expression (STAR methods; Table S5). To narrow the search, we intersected these 455 genes with those differentially expressed between deceased and other patients, resulting in a final set of 107 candidate genes (Table S5). Among these, genes contained on the MYCN amplicon *MYCN*, *NBAS*, and *DDX1* showed a positive ASE-expression effect due to strong upregulation by mono-allelic amplifications. In contrast, chromosome arm 1p (56%) and 17p (12%) were most frequent among all 76 genes with negative ASE-expression effect, indicating that loss of 1p and 17p underlies downregulation of these genes in tumors of deceased patients.

Interestingly, a substantial negative ASE-expression association was found in the Retrotransposon Gag-like 1 (*RTL1*) gene, which was upregulated in tumors of deceased patients (Figures 5C and 5D). *RTL1* is a maternally imprinted gene involved in placental/neonatal development^74^ and widely expressed in the nervous system.^75^ Upregulation of *RTL1* confers selective growth advantage in hepatocarcinoma^76^ and promotes cell proliferation by regulating Wnt/β-Catenin signaling in melanoma.^77^ *RTL1* was one of 16 genes informative for survival time in a previous study of high-risk neuroblastomas, with stronger *RTL1* expression associated with shorter survival.^78^ Our linear model revealed only a minor contribution of SCNAs and germline variants to ASE in *RTL1* (Figure S23), suggesting that differences in allelic expression levels may result from methylation differences. Analyzing a subset of tumors using bisulfite sequencing (BS-seq) (STAR methods), we found that decreased methylation levels at CpGs upstream of *RTL1* are associated with higher *RTL1* expression (Figures 5E, 5F, and S24). Taken together these findings suggest that upregulation of *RTL1* in neuroblastoma is induced by bi-allelic activation in tumors with unfavorable prognosis, likely due to loss of imprinting on the maternal allele (Figure 5G).

## Discussion

We here systematically characterized the effects of copy-number dosage on neuroblastoma gene expression and demonstrated how copy-number gains interact with upregulated *TERT* to increase the efficacy of CTM. We found 11q loss and strong 17q gain as markers of ALT in addition to *ATRX* alterations, and revealed upregulation of histone variant genes *H3F3B*, *H3F3C*, and *H2AFJ*.

Histone variants replace replication-dependent canonical histones in nucleosomes during the cell-cycle, affecting chromatin organization at telomeric,^79^ actively transcribed regions by replication-independent chromatin incorporation^80^^,^^81^^,^^82^ and interaction with chaperones and chromatin factors.^83^ *H3F3B* resides on 17q, and our findings strongly suggest that *H3F3B* is directly upregulated by 17q gains, which have already been reported to exert oncogenic effects through increased gene dosage.^43^ In contrast, *H3F3C* and *H2AFJ* expression are associated with 11q loss and 17q gain, but neither of these reside on these chromosome arms, suggesting possible involvement of regulatory effects in *trans*.

Possibly, this way copy-number alteration mediate histone replacement and chromatin re-organization in ALT, leading to decondensation and increased transcription.^80^^,^^81^ Dosage-dependent downregulation of the repressive PRC2/*EED*-*EZH2* complex, which methylates the lysine 27 residue of H3 histones may contribute to this reprogramming, and we found *EED*, which is predicted to interact with all three histone variants, as differentially downregulated in ALT tumors by 11q loss. Similarly, PRC2 activity in pediatric high-grade glioma is impaired by H3.3K27M mutations altering *EZH2* binding^67^ and resulting in depletion of H3K27 di- and tri-methylation.^66^

It is important to note that 17q gain is the single most prevalent cytogenetic feature across neuroblastoma, found in 91% (105/115) tumors of our cohort. However, we found the strongest 17q gains in the ALT group, even after controlling for covariates including tumor purity. 40–50% of variance in H3F3B gene expression was explained by copy-number differences and expression was again significantly higher in ALT tumors.

*ATRX* stabilizes telomeres through depositing of H3.3 histones, thereby preventing replication-induced breaks conducive to ALT.^79^^,^^84^ In contrast, *ATRX* is not required to deposit H3.3 histones in actively transcribed regions.^79^ Consequently, H3.3 upregulation through *H3F3B* dosage in ALT tumors with defective *ATRX* may increase the prevalence of H3.3 in nucleosomes of active chromatin without its stabilizing effect at telomeres. Importantly, we found 11q loss and 17q gain to be associated with ALT independent of *ATRX* mutations. Because not all ALT tumors harbor *ATRX* alterations, deregulated histone variants may contribute to the ALT phenotype more directly. In high-grade gliomas ALT frequently occurs in H3.3G34R-mutant tumors independent of *ATRX* alterations,^28^ indicating a functional link between impaired H3.3 function and ALT.

Additionally, loss of *ATRX* alone may not be sufficient to induce ALT,^84^ and *ATRX* mutations are likely not the only molecular feature responsible for this phenotype. However, in *ATRX-*wild type ALT-positive neuroblastomas, *ATRX* protein levels were found to be significantly decreased,^31^ suggesting that impaired *ATRX* activity could still underlie ALT in these cases. Furthermore, not all ALT-positive tumors showed 11q loss and strong 17q gain and these alterations were also present in a few ALT-negative tumors. Generally, ATRX can stabilize replication forks and is involved in the resolution of G-quadruplex DNA structures.^85^ Impaired ATRX activity could still cf. phenotypes independent of histone variant deregulation. Additional research with larger cohorts will be needed to further characterize the relationship between histone variants and ALT in neuroblastoma.

In addition to upregulated histone variants we also identified 17q copy-number dependent upregulation of predicted *ATRX* interaction partner *RAD51C*, a recombinase of the HRR pathway. *RAD51* is involved in C-circle-associated *RAD52*-independent ALT-mediated telomere synthesis.^86^ However, a previous study found that depletion of *RAD51* and its homologs including *RAD51C* had no marked effect on telomere length, fragility, or APB formation in mouse embryonic fibroblasts lacking telomerase.^87^ This either suggests that *RAD51C* may not be essential for ALT or that, unlike the model, ALT-mediated telomere synthesis in neuroblastoma favors the *RAD51*-associated and *RAD52-*independent pathway, further supported by high prevalence of C-circles in ALT neuroblastomas,^31^ as well as the prognostic value of and chemotherapy resistance conferred by *RAD51* expression in this disease.^88^

In tumors with a 17p LOH event, loss of function of *TP53* due to a second hit could be responsible for the poor prognosis of these tumors, but no such second hit was found in our cohort and we did not observe a copy-number dosage effect on *TP53* expression. Survival-associated 17p copy-number dosage effects were enriched for neuronal genes, suggesting a potential impairment of neuronal processes. However, the exact mechanism that underlies higher mortality of donors with 17p imbalance still needs to be investigated.

Lastly, we identified *RTL1* as a candidate marker for tumors with unfavorable prognosis due to loss of imprinting of the maternal allele, similar to earlier reports on loss of imprinting of the *IGF2* gene in Wilms’ tumors.^89^ The transposon-derived *RTL1* gene^90^ is part of a broader imprinted *DLK1*-*DIO3* gene cluster with three paternally expressed genes *DLK1*, *RTL1*, and *DIO3*. *DLK1* expression in neuroblastoma cell lines is associated with neuroendocrine lineage differentiation.^91^ Possibly, imprinting heterogeneity at the *DLK1*-*DIO3* gene cluster reflects differentiation states in neuroblastoma progenitor cells, and incomplete imprinting may characterize the cell-of-origin of more aggressive or treatment-resistant tumors.

Taken together, our analyses shed light on the complex interaction of genetic, epigenetic, and transcriptomic effects and how gene dosage interacts with other genetic and epigenetic factors to shape the regulatory landscape of neuroblastoma. In the future, the increase in size of such multi-omics datasets will enable a more complete understanding of the development of disease-relevant phenotypes and potentially convergent pathways.

### Limitations of the study

Neuroblastoma is a rare childhood cancer with an incidence as low as 11 cases per million children.^92^ As such, sample sizes of genomics cohorts, in particular with WGS data, remain limited. In addition, the systematic laboratory evaluation of telomere maintenance mechanisms are rarely routinely performed and often difficult to establish post-hoc. We here employ a combination of k-mer counts, TERT rearrangement and expression, and MYCN amplification status to determine telomere maintenance mechanisms. We validated this approach against the experimentally determined status of APB^21^ and C-circles^31^ in subsets of analyzed tumors with great success. Further experimental validation is crucial for a deeper understanding of the role of histone variants in maintaining ALT, particularly the histone H3.5 and H2A.J variants, whose connection to ALT remains unknown. However, this investigation was beyond the scope of the current study.

## Resource availability

### Lead contact

Further information and requests for resources and reagents should be directed to and will be fulfilled by the lead contact, Roland F Schwarz (roland.schwarz@iccb-cologne.org).

### Materials availability

This study did not generate new unique reagents.

### Data and code availability

• The raw data analyzed in this study is available from the European Genome-phenome Archive (https://www.ebi.ac.uk/ega/) under accession number EGAS00001001308,^20^ EGAS00001004022,^8^ EGAS00001005604^93^ and EGAS00001006983. These datasets are available under restricted access due to data privacy laws. Access to the EGA archive datasets is obtained by formal application to the corresponding Data Access Committee (DAC).
• Processed data and analysis code is available in Zenodo under DOI https://doi.org/10.5281/zenodo.8373208.
• Any additional information required to reanalyze the data reported in this paper is available from the lead contact upon request.

## Acknowledgments

This study was supported by funding from the Berlin Institute of Health project “TERMINATE-NB” (CRG04) to A.E. and U.O., as well as DFG research training group “GRK 1772: Computational Systems Biology” (project number 191312833) to M.B. and U.O. We thank the patients and their parents for granting access to the tumor specimens and clinical information that were analyzed in this study. R.F.S. is a Professor at the Cancer Research Center Cologne Essen (CCCE) funded by the Ministry of Culture and Science of the State of North Rhine-Westphalia. This work was partially funded by the German Ministry for Education and Research as BIFOLD—Berlin Institute for the Foundations of Learning and Data (ref. 01IS18025A and ref. 01IS18037A). The authors would like to thank the Helmholtz Association, Germany, for support and Martin Peifer for helpful comments and suggestions. Computation was performed on the HPC for Research cluster of the Berlin Institute of Health.

## Author contributions

M.B., A.E., J.S., S.M.W., D.B., R.F.S., and U.O. contributed to the study design and the collection and interpretation of the data. E.B. and N.T. performed quality control of sequencing data and aligned WGS and RNA-seq reads. M.B. analyzed WGS and RNA-seq data. M.B. performed allele-specific expression and allele-specific copy-number analysis. E.B., N.T., and M.B. analyzed somatic SNVs. J.T. and M.B. analyzed somatic SVs. R.M. and M.B. performed QTL mapping. M.B. conducted telomere length analysis. M.B. and C.W. conducted copy-number-survival analysis. M.B., R.F.S., and U.O. wrote the manuscript. J.S., R.F.S., and U.O. led the study design.

## Declaration of interests

The authors declare no competing interests.

## STAR★Methods

### Key resources table

**Table:** 

REAGENT or RESOURCE	SOURCE	IDENTIFIER
**Deposited data**		

Whole-genome sequencing data of primary neuroblastoma	European Genome-phenome Archive (EGA)	EGAS00001001308
Whole-genome- and RNA-sequencing data of primary neuroblastoma	European Genome-phenome Archive (EGA)	EGAD00001005488
Variant call files	1000 Genomes Project (phase 3)^93^	https://www.internationalgenome.org/category/phase-3/
Mappability track	UCSC Genome Browser^94^	wgEncodeCrgMapabilityAlign50mer https://genome.ucsc.edu/
Processed data and analysis code	–	https://doi.org/10.5281/zenodo.8373208

**Software and algorithms**		

Telseq v0.0.2	Ding et al.^38^	https://github.com/zd1/telseq
Bcftools v1.8	–	https://www.htslib.org/
ASCAT v2.6	Loo et al.^95^	https://github.com/VanLoo-lab/ascat/
HTseq v0.9.1	Putri et al.^96^	https://github.com/htseq/htseq
DESeq2 v1.26.0	Love et al.^97^	https://github.com/htseq/htseq
Ensembl variant effect predictor (VEP) v101.0	McLaren et al.^98^	https://www.ensembl.org/info/docs/tools/vep/index.html
BWA-MEM v0.7.15	Li and Durbin^99^	https://github.com/lh3/bwa
STAR v2.5.3a	Dobin et al.^100^	https://github.com/alexdobin/STAR/releases
GATK/Mutect2 v2.2	McKenna et al.^101^	https://gatk.broadinstitute.org/
Novobreak v1.1.3	Chong et al.^102^	https://sourceforge.net/projects/novobreak/
MEDICC2	Kaufmann et al.^40^	https://pypi.org/project/medicc2/0.5b3/
fgsea v1.12.0	Korotkevich et al.^103^	https://bioconductor.org/
GATK/ASEReadCounter v3.5.0	McKenna et al.^101^	https://gatk.broadinstitute.org/
PEER	Stegle et al.^104^	https://github.com/PMBio/peer/
FastLMM v0.2.23	Lippert et al.^105^	https://github.com/fastlmm/FaST-LMM
STRING DB network viewer v11.0b	Szklarczyk et al.^106^	https://string-db.org/
bcl2fastq v.2.19.0.316	–	https://support.illumina.com/sequencing/sequencing_software/bcl2fastq-conversion-software.html
PiGx BS-seq pipeline	Wurmus et al.^107^	https://github.com/BIMSBbioinfo/pigx_bsseq
FastQC v0.11.9	–	https://github.com/s-andrews/FastQC
TrimGalore v.0.6.2	–	https://github.com/FelixKrueger/TrimGalore
bwa-meth v.0.7.17	Pedersen et al.^108^	https://github/com/brentp/bwa-meth/
samblaster v.0.1.24	Faust et al.^109^	https://github.com/GregoryFaust/samblaster
methylKit v1.15.4	Akalin et al.^110^	https://github.com/al2na/methylKit

### Experimental model and study participant details

This study is based on the analysis of sequencing data from tumor and blood samples of patients diagnosed with neuroblastoma between 1991 and 2016. Patients were registered and treated according to trial protocols of the German Society of Pediatric Oncology and Hematology (GPOH). Samples were collected at diagnosis from untreated patients. The study was conducted in accordance with the World Medical Association Declaration of Helsinki (2013) and good clinical practice. Informed consent was obtained from all patients or their guardians. Collection and use of patient specimens was approved by the institutional review boards of Charité Universitätsmedizin Berlin and of the Medical Faculty, University of Cologne. Collected specimens and clinical annotations were archived and made available by Charité-Universitätsmedizin Berlin or the National Neuroblastoma Biobank or or the National Neuroblastoma Biobank and Neuroblastoma Trial Registry (University Children’s Hospital Cologne) of the GPOH.

### Method details

#### Whole-genome- and RNA-sequencing data

Sequencing data were collected from tumor and blood samples of patients diagnosed with neuroblastoma enrolled and treated according to trial protocols of the German Society of Pediatric Oncology and Hematology (GPOH) in the multi-center study GPOH-NB2004.^37^ Analyzed DNA and RNA samples were obtained from primary tumors of at least 60% tumor cell content as evaluated by a pathologist. *MYCN* copy-number was determined by FISH. DNA was extracted from fresh-frozen tumor tissue and the corresponding matched normal using the Puregene Core Kit A (Qiagen) and NucleoSpin Blood DNA extraction kit (Macherey-Nagel), according to the manufacturers’ instructions. Libraries were prepared with the TruSeq DNA PCR-free sample preparation kit (Illumina). WGS of tumor-normal pairs was performed on the HiSeq X-Ten platform (Illumina, San Diego, USA), yielding paired-end reads of 2 × 150 bp length. RNA was isolated with Trizol according to the manufacturer’s protocol (Thermo Fisher). Purity was analyzed on a Nanodrop 2000 spectrometer and RNA integrity assessed on a Bioanalyzer 2100 or TapeStation4200 as per manufacturer’s instructions. Only samples with an RNA integrity number of 8 or above were included. Depletion of ribosomal RNA (rRNA) was performed by enzymatic digestion. Ribo-depletion of 95–99% was confirmed using RT-qPCR. The ribo-depleted RNA was used for generation of RNA sequencing libraries using the TrueSeq Stranded mRNA kit according to the manufacturer’s protocol (Illumina). Ribo-depleted RNA was sequenced on the HiSeq4000 platform (Illumina, San Diego, USA) yielding reads of 2 × 150 bp length. Additional sequencing data were obtained from the European Genome-phenome Archive under accession number EGAS00001001308 for a non-overlapping set of donors from a previous study on somatic structural rearrangements in neuroblastoma.^20^ After quality control 52 donors of this study were included, yielding a total of 115 donors with matched tumor RNA-seq, tumor WGS, and blood-derived normal WGS.

#### Whole-genome bisulfite sequencing data

Whole-genome bisulfite sequencing data was obtained for a subset of 23 tumors (Table S1). Libraries were prepared using the EpiTect Bisulfite and Illumina Truseq PCRFree DNA sequencing kit (V2.5) and sequenced on the Illumina Hiseq X platform yielding paired-end reads of 2 × 150 bp.

### Quantification and statistical analysis

#### Whole-genome- and RNA sequencing read alignment

Reads were aligned to the GRCh37. WGS reads were aligned with BWA-MEM 0.7.15.^94^ RNA-seq reads were aligned with STAR 2.5.3a.^95^ Samblaster 0.1.24^96^ was used to mark duplicates in alignment files. Quality control was performed using FastQC. Table S1 lists donors from which matched tumor and normal WGS as well as matched tumor RNA-seq was used in the analyses.

#### Gene expression quantification

Aligned tumor RNA-seq reads were counted using HTseq/htseq-count 0.9.1 on exons of protein coding genes according to Ensembl release 75 human gene annotations for the GRCh37 reference, summarizing counts on gene-level. We normalized gene expression for the purpose of between-sample comparisons in a given gene. To mitigate the effect of sequencing depths and batch effect introduced by different RNA library preparation- and sequencing methods between the two cohorts we normalized htseq counts by the following strategy: We first calculated library-size normalized DESeq2 variance stabilized counts from htseq counts. Then, we modeled the variance stabilized counts by cohort membership using a linear model for each gene and determined the residual for each gene and sample. If not indicated otherwise, this residual was used as the measure for gene expression in our analyses.

#### Somatic single nucleotide and structural variation calling

Somatic SNVs were called by Mutect2 version 2.2 from the GATK software package.^97^ SNV calls were filtered using a panel of normals. Effects of SNVs were predicted using the Ensembl variant variation effect predictor version 101^98^ in offline mode with distance 100,000 bp. SNVs in categories missense, splice, stop, synonymous, 5′ UTR and 3′ UTR were summarized to gene level somatic mutation burden. Somatic SNVs annotated as promoter variants by the Ensembl variant effect predictor were considered separately. Splice, nonsense and missense variants for each gene were summarized based on the assigned consequence.

SV were called using novobreak version 1.1.3^99^ in pairs of matched tumor and normal WGS alignments. We only kept SV calls with QUAL ≥30, at least 5 high quality reads in support of each breakpoint in the tumor sample, 0 reads supporting each breakpoint in the normal sample, 5 or more discordant reads per breakpoint in the tumor sample and 3 or less discordant reads per breakpoint in the normal sample. The functional effects of SVs at the *TERT* locus have been established previously^20^^,^^33^ and for the detection of *TERT* SVs we relaxed the threshold on high quality reads in support of each breakpoint, requiring at least 2 of those reads to keep the SV call. Other thresholds were applied as described above. *TERT* rearrangement status was assigned to a sample positive for at least one somatic SV 100,000 kb upstream or downstream of *TERT* gene start and end coordinates (Ensembl/GRCh37) or annotated as *TERT* rearranged in.^20^ Additionally, the *TERT* locus was manually examined in tumor and normal WGS alignments for SVs that were missed by the variant calling procedure above, resulting in one additional assignment of positive *TERT* rearrangement status in tumor CB2064 (Figure S25).

We used a targeted approach to identify *ATRX* exon deletions. To this end we determined read coverage at *ATRX* gene coordinates in 50 bp bins, normalized the read counts by the number of overall mapped reads and defined a tumor coverage ratio by s_i_ = log2(*n*_*i*T_/*n*_*i*N_), where *n*_*i*T_ and *n*_*i*N_ are normalized read counts in tumor and normal for bin *i* respectively. For each matched tumor/normal pair we then fit a two-component Gaussian mixture model to the signal and determine the mean and relative proportions of two hypothetical clusters, corresponding to read coverages of deleted and intact regions of the gene. Samples that harbored a signal mean difference of at least 1.5 units between the two clusters and in which the smaller cluster showed a proportion of 10% or more of the larger cluster were regarded as *ATRX* deleted. Tumors that showed either *ATRX* deletions as determined by this method, were positive for a somatic SV breakpoint inside *ATRX* gene boundaries or carried a somatic missense, nonsense or splice SNV were considered as mutant *ATRX*.

#### Telomere maintenance analysis

Telomere lengths were estimated from WGS of normal and tumor samples by Telseq 0.0.2^38^ with parameter -u (ignore read groups) and otherwise default settings. Briefly, the method estimates telomere length by counting WGS reads containing the telomere repeat sequence (TTAGGG)^*k*^, where *k* denotes the number of repeats of the 6-mer. Telseq uses default repeat length k = 7 and normalizes the resulting read count by GC content and a genome size factor. The authors calibrated the default parameters using telomere length measurements determined by Southern blot analysis of terminal restriction fragments. We summarized telomere lengths per sample by the log telomere length ratio log(TLR) = log(L_T_/L_N_), where L_T_ and L_N_ are the Telseq estimates for telomere length in tumor and normal WGS sample respectively.

Tumors were clustered based on unsupervised Gaussian mixture modeling of *TERT* gene expression (*n* = 2 mixture components). A threshold (*Z* score > −0.1028) for high *TERT* expression was defined as the *TERT* expression *Z* score at which the probability of assignment of a tumor to the component of stronger *TERT* expression exceeded 95%, similarly as described in.^21^ We assigned the *TERT-high* attribute to tumors that exceeded the *TERT* expression threshold and for which neither *MYCN* amplification nor *TERT* rearrangements were detected.

The telomere maintenance status (CTM, ALT, Mix, or None) was assigned based on the status of *MYCN* amplification, *TERT* rearrangement, *TERT-high* attribute and telomere length ratio as follows: Status *canonical telomere maintenance* (CTM) was assigned to tumors with either *MYCN* amplification, *TERT* rearrangement or that were classified as *TERT-high*. Status *alternative lengthening of telomeres* (ALT), was assigned to tumors with telomere length ratio log(L_T_/L_N_) > 0.5. Status *Mix* was assigned to all tumors that met criteria for both CTM and ALT. Status *None* was assigned to all other tumors, indicating a general lack of evidence of any telomere maintenance mechanism.

#### Genotyping and phasing

Variant call files with 84,801,880 germline variants from the 1000 Genomes Project (phase 3)^100^ were downloaded and filtered for biallelic SNPs. SNPs from chromosomes 1–22 were filtered for ≥1% minor allele frequency (MAF) in the 1000 Genomes cohort. Only SNPs with a mappability score of 1 (unique 50mer, UCSC wgEncodeCrgMapabilityAlign50mer)^101^ were kept, resulting in 9,866,569 variant sites (*SNP panel*) for downstream analysis. We generated pileups at positions of the SNP panel from WGS alignments of blood-derived control samples by Bcftools 1.8 mpileup, excluding unmapped reads, or reads that were marked as optical duplicates or “not primary alignment”. The resulting pileups were used as input to the Bcftools 1.8 multiallelic-caller to call genotypes at the positions of the SNP panel. We only kept resulting genotypes with an allelic depth of 10 or more reads and a genotype quality of 20 or higher. The resulting individual variant files were merged and genotypes were phased by Eagle 2.4^102^ using the phased 1000 Genomes genotypes as reference. The resulting variant file, comprising phased genotypes of all individuals was defined as the *genotype panel* for further downstream analysis.

#### Copy-number analysis

Pileups of primary tumor WGS were generated by Bcftools 1.8 mpileup at SNP positions of the genotype panel established (STAR methods). Unmapped reads, or reads that were marked as optical duplicates or as “not primary alignment” were not considered in the pileup. For each of the SNPs the allelic depths were calculated from the pileups on normal and tumor alignments respectively. For SNPs with total depth of 10 or more reads in both tumor and normal alignments we determined the B-allele frequency (BAF) and the coverage log ratio (LogR). For a given pileup position the BAF is defined as the ratio between alternative allele nucleotide count and the number of total considered counts a_i_/(r_i_+a_i_), where a_i_ and r_i_ are the allelic depths of alternative and reference allele respectively. The LogR at SNP position *i* was defined as log2((d_ti_/d_ni_)/(∂_t_/∂_n_)), where d_ti_ is the total depth at SNP position *i* in the tumor sample, d_ni_ is the total depth at SNP position *i* in normal sample and ∂_t_ and ∂_n_ are mean depths at SNPs of tumor and normal sample respectively.

The BAF of a heterozygous SNP position is informative for the proportion of aligned reads originating from the paternal and maternal allele. At a homozygous SNP the BAF is expected to be close or equal to 1, if the sample’s SNP genotype is homozygous alternative or close or equal to 0 if the genotype is homozygous reference. The BAF is calculated separately for alignments of normal and tumor, resulting in a *normal BAF* and a *tumor BAF* per SNP and sample. The LogR is a measure of total coverage difference between normal and tumor samples and is informative at any position, including homozygous and heterozygous SNPs. It is calculated for a pair of alignments (tumor and normal), resulting in a LogR value per SNP and sample.

Allele-specific copy-number profiles were generated from tumor and normal BAFs and LogR values for each sample using ASCAT 2.6^103^ with a custom segmentation procedure. In ASCAT’s segmentation step the BAF and LogR values are converted into intervals of similar values. ASCAT’s original implementation of this segmentation considers both LogR and BAFs to obtain start and endpoints for segments. We found noisy coverage log ratios to introduce over-segmentation in some samples and therefore replaced the segmentation procedure with a custom implementation that only considers BAFs to determine start and endpoints of segments, but still estimates the segment’s coverage using the log coverage ratios. ASCAT’s output comprises copy-number segments with integer copy-numbers of major and minor alleles as well as estimates for tumor purity and ploidy. All copy-number segments were inspected manually for quality. For samples with estimated tumor purity less than 60% copy-number calling was rerun with adjusted purity and ploidy values that were manually selected after inspection of the goodness-of-fit plots and in agreement with pathology estimates of tumor purity.

We associated the copy-number per chromosome arm with telomere length. For this purpose we derived a copy-number LogR per chromosome arm and tumor by an overlap length-weighted mean considering all copy-number segments of a given sample overlapping a chromosome arm. Samples were divided into two groups (ALT, non-ALT), excluding a single tumor (NBL54) with signs of both ALT and canonical telomere maintenance by *TERT* rearrangement (group *Mix*). We then used this binary (ALT, non-ALT) outcome as the response of a generalized linear model controlling for covariates *MYCN* amplification, *ATRX* alteration, age, sex, cohort, tumor purity, and tumor ploidy. The association *p*-value was determined by an analysis of variance (ANOVA) using a Chi-Squared test between two generalized linear models, of which the first modeled ALT by the covariates above and the second additionally included a term for the copy-number LogR. P-values were determined for each chromosome arm and corrected by the Bonferroni method. Chromosome arms below 0.05 FWER were considered significant.

#### Estimation of whole-genome-doubling

The WGD (whole-genome-doubling) status of the samples were estimated using the phylogenetic reconstruction tool MEDICC2.^40^ For single samples MEDICC2 calculates the minimum number of evolutionary events from a diploid genotype to the copy-number states of the sample by combining loss-of-heterogeneity events, whole-genome-doubling as well as chromosome-wide and focal losses and gains. By checking whether the shortest evolutionary path from the diploid to a sample copy-number profile contains a WGD we can estimate the WGD status. As a WGD event followed by multiple losses can also be modeled by multiple gains, MEDICC2 can be conservative in its WGD estimation. We employed a bootstrap method to improve the estimation of WGD events. For this we used 100 bootstrap copy-number profiles per sample that were created by randomly drawing 22 chromosomes with replacement from the original chromosomes. If 5% or more of the bootstrap runs exhibited a WGD the sample was marked as WGD positive.

#### Classification of copy-number states

Copy-number states (CN state) were assigned to each segment based on ASCAT’s allele counts, ploidy estimates and LogR of coverage between tumor and normal WGS alignment. Copy-number (CN) states were assigned based on conditions in the following order: *weak gain*: CN_total_ > round(ploidy); *medium gain*: CN_total_ > 1.5 × round(ploidy); *strong gain*: CN_total_ > 2.5 × round(ploidy); *shallow loss*: CN_total_ < round(ploidy); *loss*: CN_total_ < 0.5 × round(ploidy). Here, CN_total_ = CN_major_ + CN_minor_, where CN_major_ and CN_minor_ are the allele counts of major and minor allele respectively and round(ploidy) the ploidy estimate determined by ASCAT rounded to an integer value. Copy-number state *amplification* was assigned to focal alterations of segments smaller than 10 Mb with CN_major_ ≥ 5 and LogR_seg_ - LogR_contig_ > 0.7, where LogR_seg_ is the mean LogR of the segment and LogR_contig_ the mean LogR of the segment’s chromosome (contig). Similarly we assigned a copy-number balance state (CN balance state) to each segment. For this purpose the copy-number ratio was determined as CN_ratio_ = CN_major_/(CN_major_ + CN_minor_). Then, the CN balance state *balance* was assigned if CN_minor_ > 0 and CN_major_ = CN_minor_. CN balance state *weak imbalance* was defined as CN_major_ > CN_minor_ and CN_ratio_ ≤ ⅔, and state *strong imbalance* was defined as CN_major_ > CN_minor_ and CN_ratio_ > ⅔. CN balance state *amplification* was defined in the same way as for the copy-number state above. Copy-number segments were marked as *LOH* if CN_minor_ = 0. We derived copy-number states for each chromosome arm and separately for cytoband 1p36, (which is frequently deleted in *MYCN*-amplified tumors) by summarizing the overlap of all segments per copy-number state in these broader regions and assigning the copy-number state of largest overlap. We applied the same procedure to assign copy-number states to genes by overlap between gene coordinates (Ensembl version 75). Gene amplification status was inferred from its copy-number state and gene-specific LogR measurements. Genes of copy-number state *amplification* or those with LogR_gene_ > 2.5 were defined as amplified, where LogR_gene_ is the mean LogR across all SNPs falling within the gene’s coordinates.

#### Allele-specific expression analysis

Allele-specific RNA read counts were determined by GATK^97^ (version 3.5.0) ASEReadCounter from RNA-seq alignments at heterozygous SNPs.^104^ SNPs with less than 8 total or less than 2 allelic reads were removed. Additionally, only sites that qualified as bi-allelic according to a statistical test were retained: A binomial test on the minimum allele count = min(alt, ref), number of trials (alt + ref) and hypothesized probability of success sum(non_ref_alt)/sum(raw_depth) was applied, where ref and alt are the reference and alternative allele counts, and non_ref_alt and raw_depth the non-reference/non-alternative allele count and raw read depth per site respectively. Sites for which the null hypothesis was rejected (FDR 0.05, Benjamini-Hochberg) were classified as bi-allelic. The reference allele bias was estimated by averaging over the reference allele fraction ref/(ref + alt) of all ASE sites from balanced copy-number regions per sample. We used statistical phasing information (see genotyping and phasing methods) to summarize allelic counts at exonic heterozygous SNPs of the same haplotype per gene. Only genes with a total of 10 or more counts from both haplotypes were retained. The ASE ratio for a given gene was calculated as max(A, B)/(A + B), where A and B are haplotype counts of the arbitrary A and B allele respectively. Expression imbalances per gene and sample were assessed by a two-sided binomial test using A as the number of successes, (A + B) as the number of trials and 0.5 as the hypothesized probability of success. The *p*-value was adjusted for multiple testing using the Benjamini-Hochberg procedure. Allelic-expression imbalance (AEI) status was assigned to observations (gene-sample pairs) for which an expression imbalance was detected at FDR 0.05.

#### *Cis*-QTL association testing

For eQTL analysis the SNP genotypes called in 115 WGS samples of normal tissue were pooled and filtered. Only SNPs with a minor allele frequency of 5% and at least 10% genotyped samples in the cohort were retained. Htseq count^105^ was used to count reads from RNA-seq data of tumor samples in the union of all exons per gene based on the Ensembl 75 gene annotation. Raw RNA gene counts were normalized by library depth per sample and transformed to variance-stabilized counts by DESeq2.^106^ Only protein-coding genes on chromosomes 1–22 with at least 10 counts in 90% of the samples were considered. In total 13,903 genes were included in the analysis. Variance-stabilized counts were centered and strong outlier samples, defined as normalized count values exceeding 3 times the standard deviation of all normalized counts per gene, were removed. To estimate the expression variability between samples we applied probabilistic estimation of expression residuals (PEER)^107^ to derive 10 factors from the normalized counts. We took these factors as representatives for global expression differences that are likely not associated with *cis*-regulatory effects and incorporated them as covariates in the association test described below. Genotypes of SNPs in a *cis*-window of 500 kb upstream and downstream of annotated gene coordinates were associated with the gene’s quantitative trait. SNPs were associated with quantitative traits by FastLMM,^108^ version 0.2.23 in single SNP mode. FastLMM uses a linear mixed model in a regression of the number of alternative alleles on quantitative phenotypes controlling for given covariates. We combined gene- and sample-specific covariates individually in each test. The somatic gene copy-number was calculated as the average total copy-number in gene intervals and used as the only gene-specific covariate. Sex, cohort, tumor purity, tumor ploidy and the 10 PEER factors were incorporated as sample-specific covariates. Each association test was controlled by the matching set of sample and sample-gene-specific covariates for a given set of gene associations.

#### Analysis of genetic effects on gene expression and ASE

We modeled both total expression and ASE by local genetic effects based on detected germline and somatic variation at the respective gene locus and additional covariates using linear regression. We predicted the ASE ratio from the lead eQTL variant (the heterozygosity status of the SNP with greatest effect size from eQTL mapping), the copy-number ratio and binary variables indicating the presence of a structural variation breakpoint overlapping with gene coordinates including +/− 100 kb flanking regions, somatic SNVs in the promoter, and at gene coordinates (including UTRs and introns) as determined by Ensembl variant effect predictor (VEP) version 101.0. Similarly, gene expression was modeled by the genotype (encoded as number of alternative alleles) of the SNP with greatest effect size from eQTL mapping, copy-number LogR, somatic structural variation and somatic SNVs in promoter and gene. Tumor purity and *MYCN* amplification status were used as additional covariates in models of both expression phenotypes. In the ASE model, the log sum of coverage at the ASE SNPs was used as an additional covariate. A linear model with up to 115 observations was fitted for each gene separately. Only genes with 20 or more complete observations (for effects/covariates and expression phenotype) were considered. The explained variance per genetic effect was determined by its relative contribution to the total sum of squares as given by ANOVA on the fitted model. Significant variance components were determined by ANOVA’s F-statistic and the resulting *p*-value was adjusted for multiple testing by the Bonferroni method for each effect. Significant effects per gene were defined as effects at 0.05 FDR.

Pathway enrichment analysis of copy-number effects on gene expression was conducted using the fgsea R package^110^ version 1.12.0 with inbuilt Reactome pathway definitions. Genes were ranked based on the variance in expression explained by copy-number effects from the variance component analysis. Significant pathways were determined at FDR <0.01. Independently enriched pathways were determined by the collapsePathways function of the fgsea R package with default parameters.

#### Association of copy-number ratio with survival

To associate allelic copy-number differences with survival we summarized copy-number ratio and LogR values in genomic regions. We calculated the copy-number ratio as CN_ratio_ = CN_major_/(CN_major_ + CN_minor_), where CN_major_ and CN_minor_, are major and minor allele counts as determined by allele-specific copy-number analysis respectively. CN_ratio_ and LogR values were summarized on the level of chromosome arms. Additionally we summarized copy-numbers in 5 Mb bins along the genome. The average value per region was defined as the mean value of CN segments overlapping the genomic region weighted by the length of overlap. 5 Mb bins overlapping amplifications were assigned the value of the amplified CN segment directly, dropping values of other segments overlapping the same bin. We used this strategy in order to maintain information about amplification of (focal) alterations in larger bins (e.g., 1 Mb focal amplification in 5 Mb bins).

We associated the summarized copy-number ratio per region with patient survival. For each region we tested for the association of copy-number ratio to survival using a generalized linear regression on the binary response “deceased from disease” vs. other. The test was set up to control for covariates *MYCN* amplification, age, tumor stage 4, sex, tumor purity and tumor ploidy. The association *p*-value was determined by an analysis of variance (ANOVA) using a Chi-Squared test. The test was carried out between a generalized linear model (GLM) of the covariates above and a second model that included the copy-number ratio in addition to these covariates. Nominal *p*-values determined for each region were corrected by the Bonferroni method and regions below 0.05 FWER were considered significant.

We used a Cox proportional hazard model^109^ to predict overall survival from the copy-number ratio of the chromosomal region identified in the regression analysis (STAR methods). In contrast to the binary survival outcome, here survival times are taken into account. Subsequent significant bins in the discovery model were merged and the average copy-number ratio was determined for the merged bins by the weighted average method as described above. Survival times were predicted by the covariates copy-number ratio, *MYCN* amplification status, age, tumor stage 4, sex, tumor purity and tumor ploidy. A survival function was estimated by the Kaplan-Meier method. Here, discretized states “balance” (copy-number ratio = 0.5) and “imbalance” (copy-number ratio >0.5) were used to split samples into two groups and to plot the corresponding survival curves.

#### Gene expression analysis

We analyzed gene expression differences between ALT and non-ALT tumors by linear regression using a similar methodology to the analysis that identified copy-number differences between these groups described above. We expect that this approach facilitates detection of expression differences mediated by the ALT-associated SCNAs identified. Expression values were modeled by linear combination of ALT status, *MYCN* amplification, *ATRX* alteration, age, sex, cohort, tumor purity and tumor ploidy. The *p*-value was derived from an ANOVA Chi-squared test for significance of the ALT status covariate and adjusted for multiple testing using the Benjamini Hochberg method. Genes with FDR <0.05 were considered as significantly different expressed in ALT tumors.

DESeq2 1.26.0^106^ was used to perform differential expression analysis on HTseq/htseq-count 0.9.1^105^ gene counts between donors marked with survival status *deceased from disease* according to the clinical annotation file and other donors on variance stabilized raw RNA-seq counts. P-values and log-fold changes of differential expression were obtained controlling for sample covariates cohort, tumor purity, age and sex. Log-fold changes were shrunken using the apeglm method.^111^ Significant genes were determined at FDR <0.05 based on the Benjamini Hochberg-adjusted *p*-value from DESeq2.

To determine genes underlying strong *cis*-regulatory control by activation or attenuation of gene expression from one of the two alleles, we performed a correlation analysis between ASE and gene expression. ASE ratios were filtered, so that only ratios from 10 or more RNA-seq read counts remained. Variance stabilized RNA-seq reads were matched with ASE ratios by sample and gene. We grouped observations by gene and only considered genes with at least 10 donors informative for ASE in that gene yielding a total of 10,862 genes with sufficient number of observations. Both ASE ratio and gene expression read counts were separately corrected by batch and tumor purity by fitting linear models per gene and obtaining residuals of expression and ASE ratio that were used in the subsequent analysis. Analogous to the ASE ratio, the *tumor DNA ratio* was defined as max(A,B)/(A + B), where A and B are phased and aggregated read counts of the tumor DNA alignment of expressed heterozygous SNPs gene for the two alleles respectively. We then modeled the ASE ratio by gene expression, cohort, tumor purity, total read count at heterozygous SNP (log) and normal DNA ratio using linear regression and determined a *p*-value for the gene expression term by ANOVA (F-statistic). Resulting *p*-values were corrected using the Benjamini-Hochberg procedure. Allelic dosage genes (AD genes) were defined as those genes at FDR <0.05. To identify a subset of AD genes with clinical relevance we matched Pearson’s r of ASE-expression correlation with adjusted *p* values from differential expression analysis. A subset of differentially expressed genes was defined by intersecting AD genes with genes significantly different expressed between deceased and not-deceased patients (FDR <0.05, Benjamini-Hochberg).

#### Protein network visualization

Protein networks visualizations were created using the STRING DB network viewer (version 11.0b, https://version-11-0b.string-db.org/).^112^ Settings were adjusted such that line thickness between nodes indicate protein interaction confidence (strength of data support) and minimum required interaction score was set to “medium” (0.4). The default set of interaction sources was used (text mining, experiments, databases, coexpression, neighborhood, gene fusion, cooccurrence).

The interaction graph in ALT associated protein interactions of ATRX and histone variant genes was subset to proteins of differentially expressed genes (ALT) (FDR <0.05) with correlation to LogR of 11q or 17q with abs(*r*) > 0.3, where *r* is Pearson’s correlation coefficient. Network nodes were colored based on up- (red) or down- (blue) regulated genes as identified in ALT differential expression analysis. Enrichment of biological processes (GO terms) in the protein network of 17p dosage effects were determined by the STRING network analysis tool.

#### TERRA analysis

To quantify telomeric RNA expression for the subset of *n* = 63 tumors of the cohort for which total RNA libraries were available, we adapted the approach of Wang et al. 2015^113^ to paired-end sequencing data. Only considering RNA fragments, for which any of the two reads contained at least five perfect consecutive matches of the telomere repeat motif TTAGGG (UUAGG), we normalized these fragment counts by total fragments counts to derive FPM (fragments per million) of telomeric RNA per tumor sample.

#### Whole-genome bisulfite sequencing analysis

Reads were extracted with bcl2fastq (v.2.19.0.316) and processed with a developer version of the PiGx BS-seq pipeline.^114^ Quality control was performed with FastQC 0.11.9. Reads were trimmed with TrimGalore v.0.6.2 and aligned to the bisulfite-converted reference human genome GRCh37 using bwa-meth v.0.7.17.^115^ Duplicate reads were removed with samblaster v.0.1.24.^96^ Germline C/T SNPs detected in our cohort at MAF >0.01 were removed using bcftools 1.9. CpG DNA methylation calling was performed with methylKit v.1.15.4^116^ at a minimum 10x coverage.

### Additional resources

The GPOH-NB2004 clinical trial is identified by EUDRAC number EU-20661 and ClinicalTrials.gov ID NCT00526318. GPOH-NB2004 clinical trial information: https://www.gpoh.de/kinderkrebsinfo/content/e1676/e9032/e68518/e206421/index_ger.html.

## Supplemental information

Document S1. Figures S1–S28

Table S1. Overview and clinical annotations, related to Figure 1C MYCN-amp: MYCN amplification; TERT-re: TERT rearrangement; TMM: telomere maintenance; CTM: Canonical telomere maintenance; ALT: Alternative lengthening of telomeres; EFS: event-free survival; OS: overall survival; TLR: telomere length ratio; CIN: chromosome instability index; WGD: whole-genome doubling; WGS: Whole-genome sequencing; WGBS: Whole-genome bisulfite sequencing; APB: ALT-associated PML-nuclear bodies.

Table S2. Survival-associated differential gene expression, related to STAR Methods and Figures S11C and S11D Results of differential expression analysis for genes in tumors of donors with survival status “deceased from disease” vs. other survival status SE: standard error.

Table S3. Reactome pathways enriched in copy-number dosage effects, related to STAR Methods and Figure S14 ES: enrichment score; NES: normalized enrichment score.

Table S4. Correlation of gene expression and 11q and 17q copy-number for differentially expressed ALT genes, related to Figures 4D and S18 Model estimate, P, and adjusted P from ALT differential expression analysis. LogR: Log-ratio (copy-number); cor.: correlation.

Table S5. Allelic dosage genes, related to STAR Methods and Figure 5C AD: allelic dosage; diff. expr.: differentially expressed.
