# Supplementary material for: Copy-number dosage regulates telomere maintenance and disease-associated pathways in neuroblastoma
Source: iScience. 2024 Sep 10;27(10):110918. doi: 10.1016/j.isci.2024.110918 (PMC11615189; doi:10.1016/j.isci.2024.110918)
Supplement: Document S1. Figures S1–S28 [file mmc1.pdf]

iScience, Volume *27*

Supplemental information

Copy-number dosage regulates telomere maintenance and disease-associated pathways in neuroblastoma

Martin Burkert, Eric Blanc, Nina Thiessen, Christiane Weber, Joern Toedling, Remo Monti, Victoria M. Dombrowe, Maria Stella de Biase, Tom L. Kaufmann, Kerstin Haase, Sebastian M. Waszak, Angelika Eggert, Dieter Beule, Johannes H. Schulte, Uwe Ohler, and Roland F. Schwarz

Supplementary figures

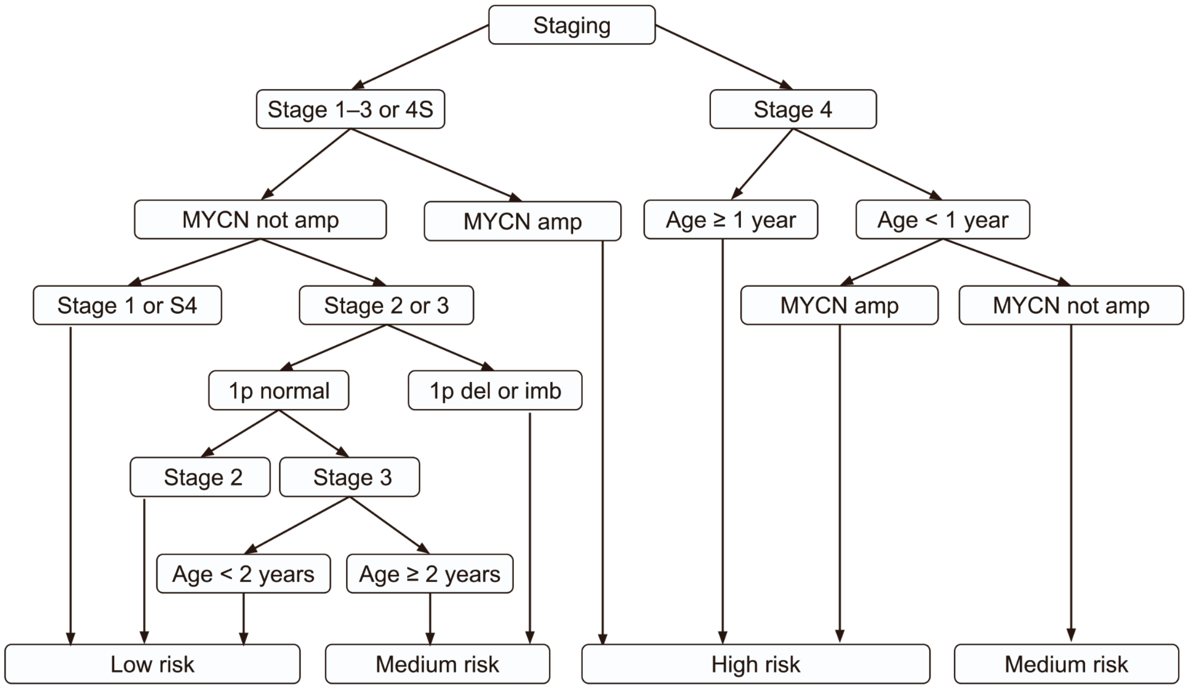

Figure S1. Risk stratification scheme of the NB2004 neuroblastoma trial, related to Figure 1C

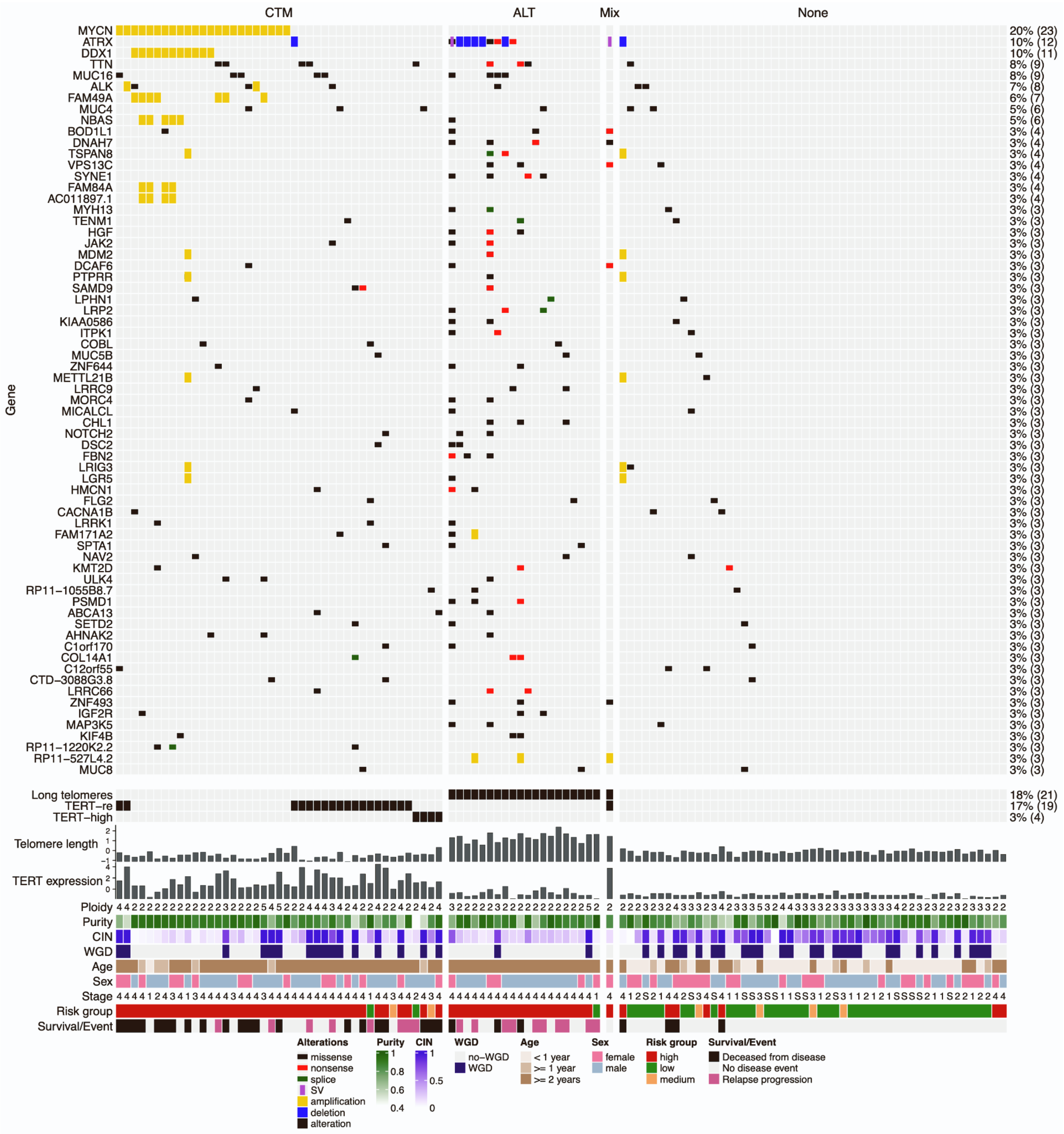

Figure S2. Frequently mutated genes in 115 primary neuroblastoma tumors by inferred telomerase maintenance mechanism, related to Figure 1C

CTM, canonical telomere maintenance; ALT, alternative lengthening of telomeres; *MYCN*-amp, *MYCN* amplification; *TERT*-re, *TERT* rearrangement; *ATRX*-mut, *ATRX* mutation.

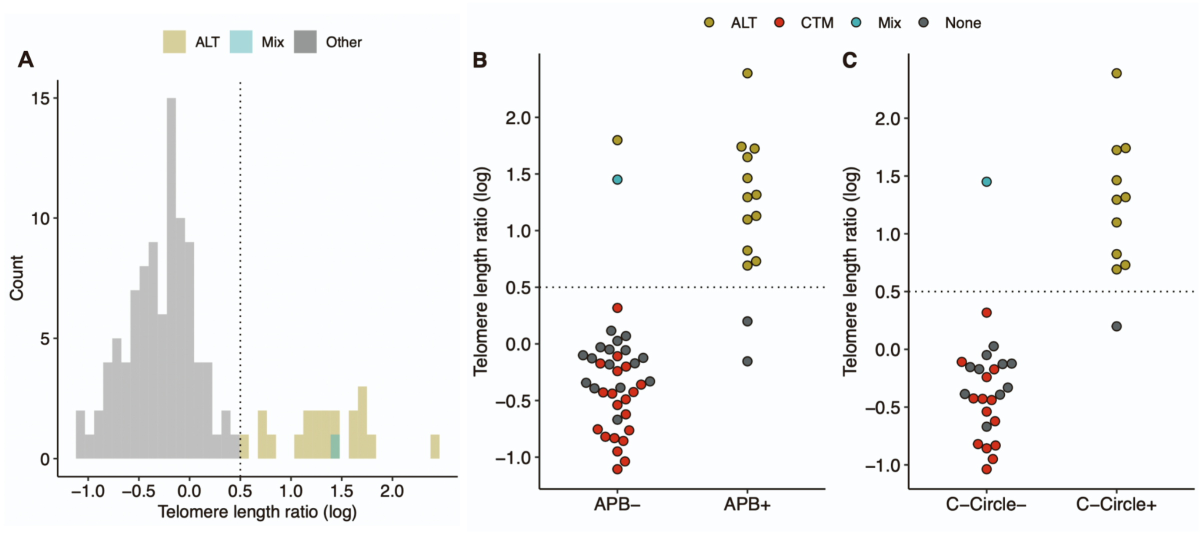

Figure S3. Telomere length ratio, ALT classification and APB status, related to STAR methods and Figure 1C

(A) Distribution of telomere length ratio (TLR) from WGS of 115 tumors/normal pairs. Color indicates inferred telomere maintenance status "ALT" and "Mix", other status in gray.

(B) Telomere length ratio by ALT-associated PML-nuclear bodies (APB)-status from ref[1] and assigned telomere maintenance group in a subset of N=52 samples analyzed.

(C) Telomere length ratio by presence of circular partially single stranded extrachromosomal telomeric repeat sequences (C-Circles) from ref[2] and assigned telomere maintenance group in a subset of N=36 samples analyzed.

Threshold [log(TLR)>0.5] as prerequisite for ALT classification shown as dotted vertical and horizontal lines in (A) and (B,C) respectively. A single sample (NBL54) was assigned telomere maintenance status "Mix" as it showed signs of both alternative lengthening of telomeres (ALT) and canonical telomere maintenance (CTM) by increased telomere length, *TERT* rearrangement and strong *TERT* expression.

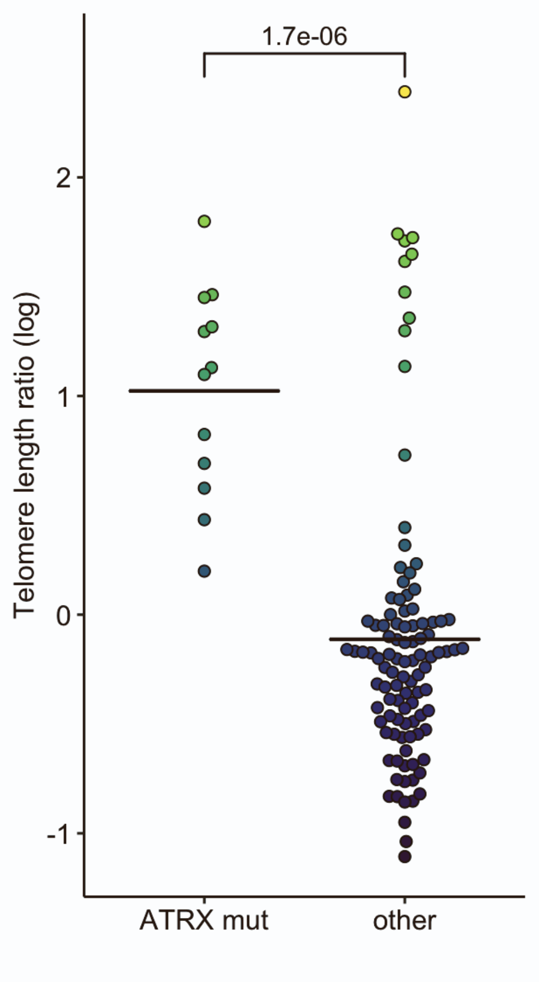

Figure S4. Telomere length and ATRX mutation status, related to Figure 1C

P-value between groups determined by one sided Wilcox rank sum test shown above bracket. *ATRX* mut, *ATRX* mutation. Horizontal bars indicate group mean.

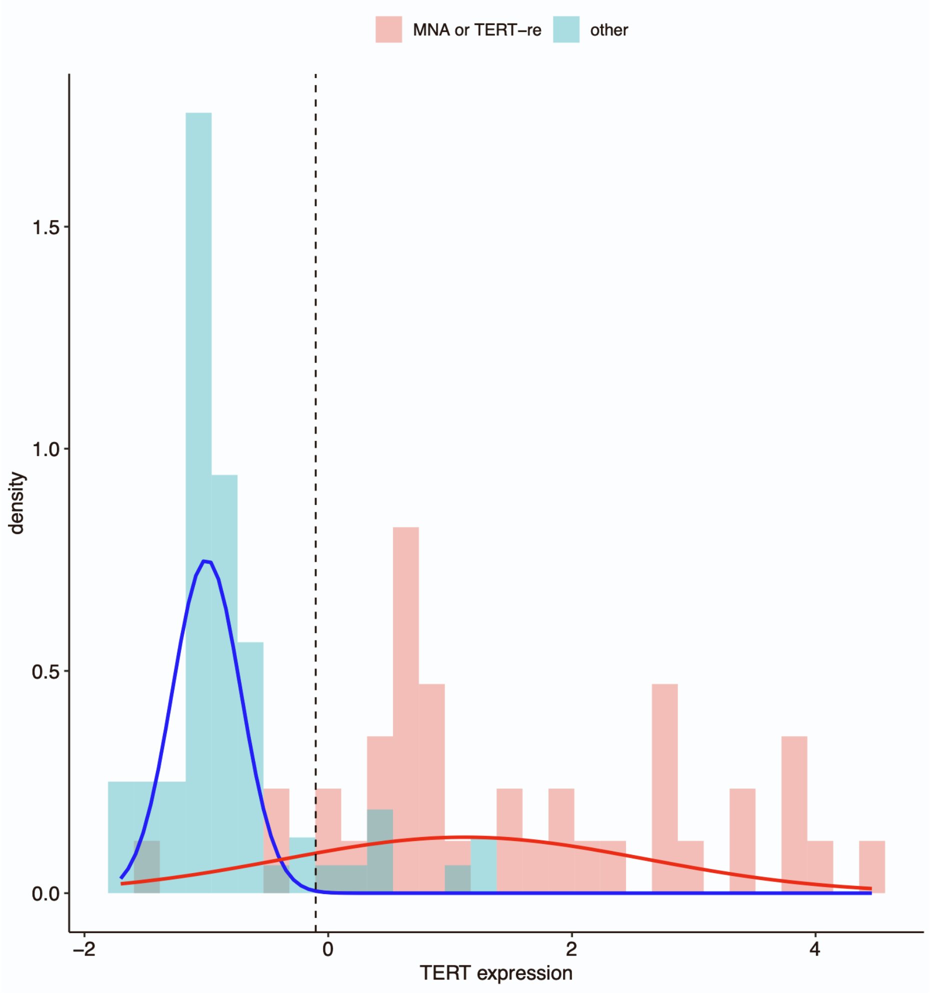

Figure S5. Distribution of TERT expression and threshold to define TERT overexpression, related to STAR methods and Figure 1C

Density curves of two components derived from unsupervised gaussian mixture modeling in blue and red. Threshold (z-score > -0.10) for high *TERT* expression indicated as vertical dashed line is defined as >95% probability for an observation belonging to the high expression component (red) similarly as described in ref [1].

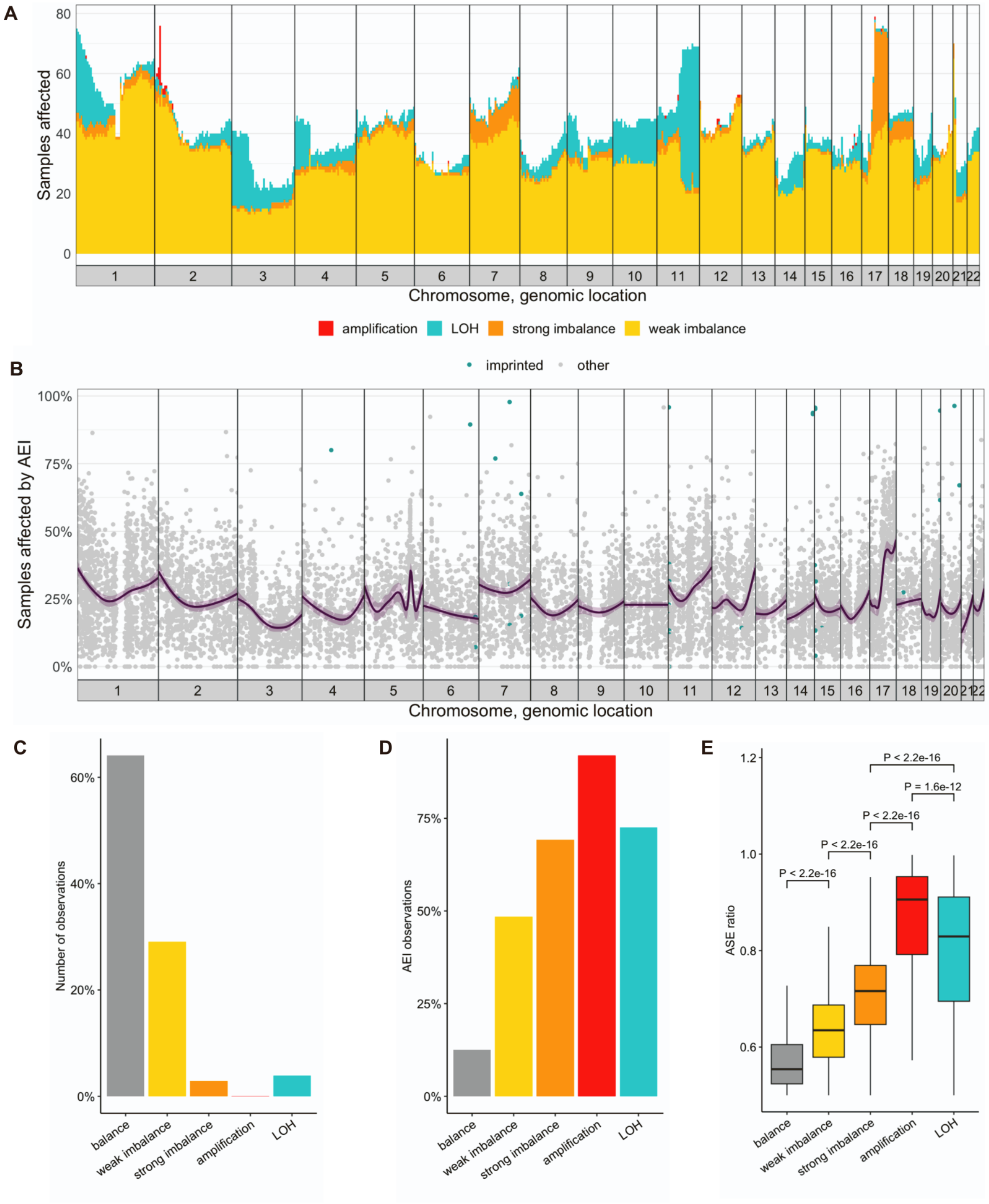

Figure S6: Genomic and expression imbalance, related to Figure 2A-C,E,G

(A) Number of samples affected by copy-number imbalances summarized in 5Mb genomic bins.

(B) AEI frequency per gene. Dark purple line: Smoothed average AEI percentage. Light purple ribbon: 95% confidence interval of average AEI percentage.

(C) Percentage of observations (gene-sample pair) per copy-number balance state. D, Percent of observations with allelic expression imbalance.

(E) Distribution of ASE ratios, outliers not shown; Midline in boxplots marks median; Upper and lower hinges mark the first and third quartile; Upper and lower whiskers extend to the smallest and largest value max. 1.5 × IQR; Wilcoxon test is shown between groups.

Gene ASE observations per copy-number balance state: balance (n = 425,082), weak imbalance (n = 193,089), strong imbalance, excl. LOH (n = 18,906), amplification, (n = 174), LOH (n = 25,934). AEI: allelic expression imbalance, LOH: loss of heterozygosity.

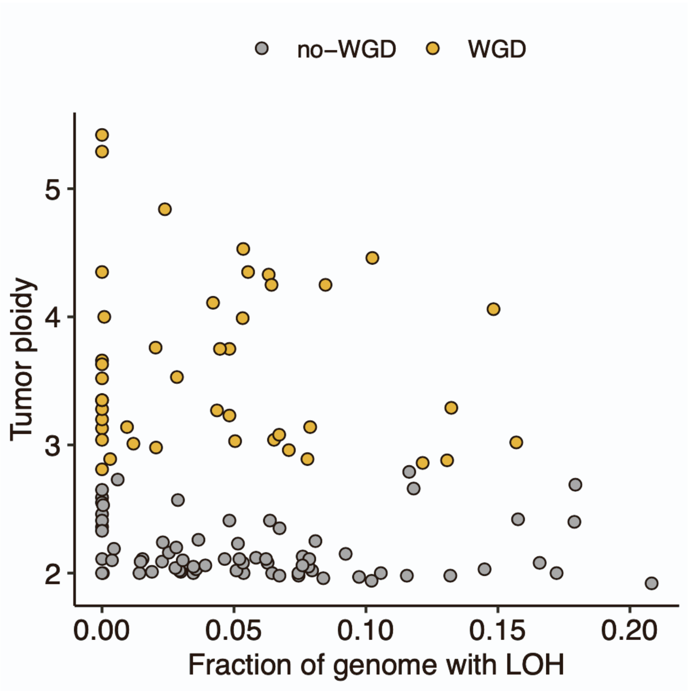

Figure S7. Ploidy and fraction of LOH per tumor indicating WGD status, related to Figure 1C and Figure 2C

WGD, Whole-genome doubling; LOH, Loss of heterozygosity.

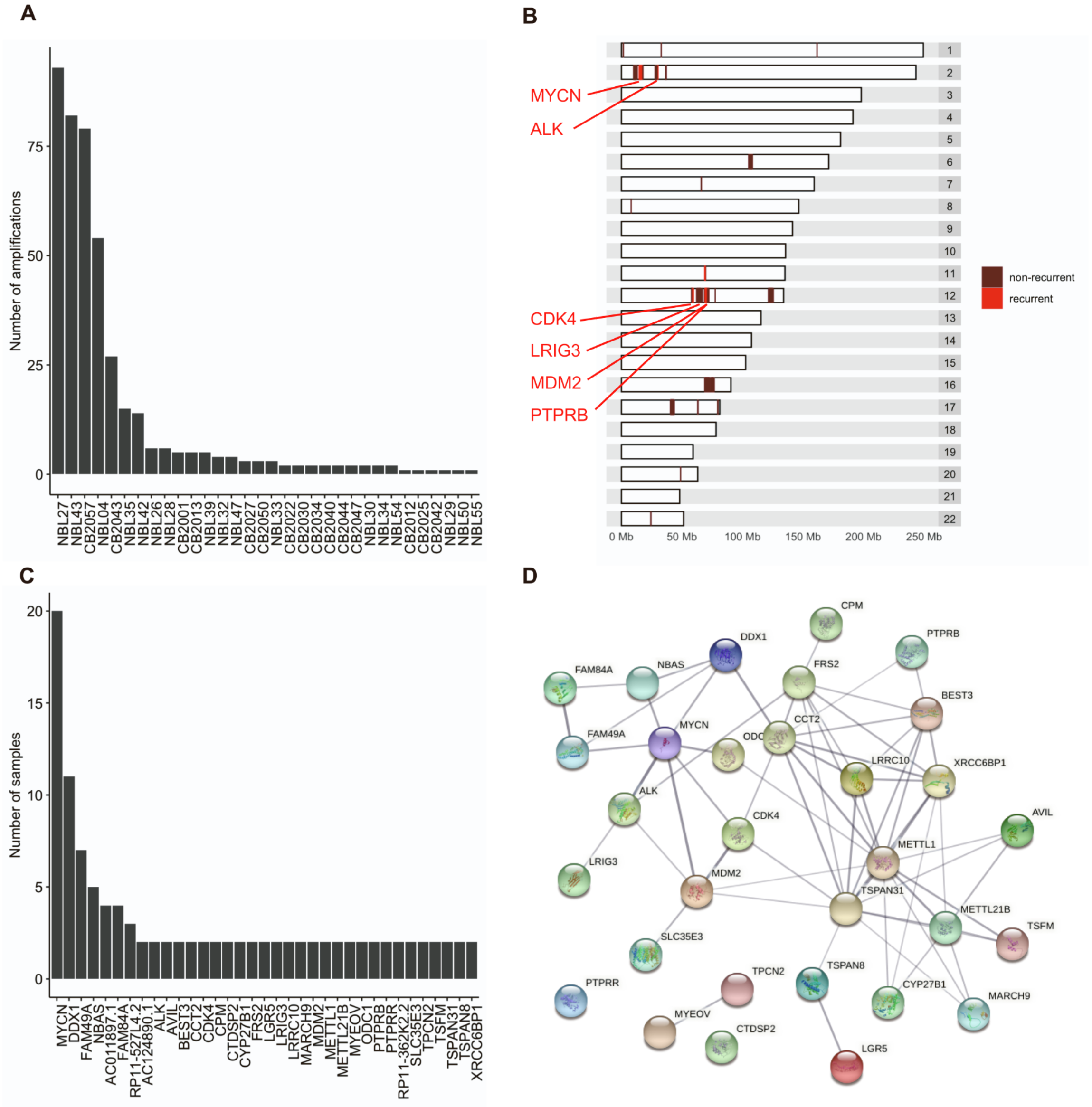

Figure S8. Recurrently amplified protein-coding genes, related to Figure 2A,H

(A) Number of amplifications per sample (samples without gene amplification are not shown).

(B) Chromosomal locations of amplified genes. Recurrently amplified COSMIC census genes are labeled by gene name.

(C) Number of samples affected by amplification in recurrently amplified genes (i.e. genes amplified in at least two samples).

(D) Network of protein interactions from the STRING database[1] for recurrently amplified genes. Thickness of edges represent data support for interactions. Network interaction enrichment $P < 1.0 \times 10^{-16}$ as given by STRING network statistics.

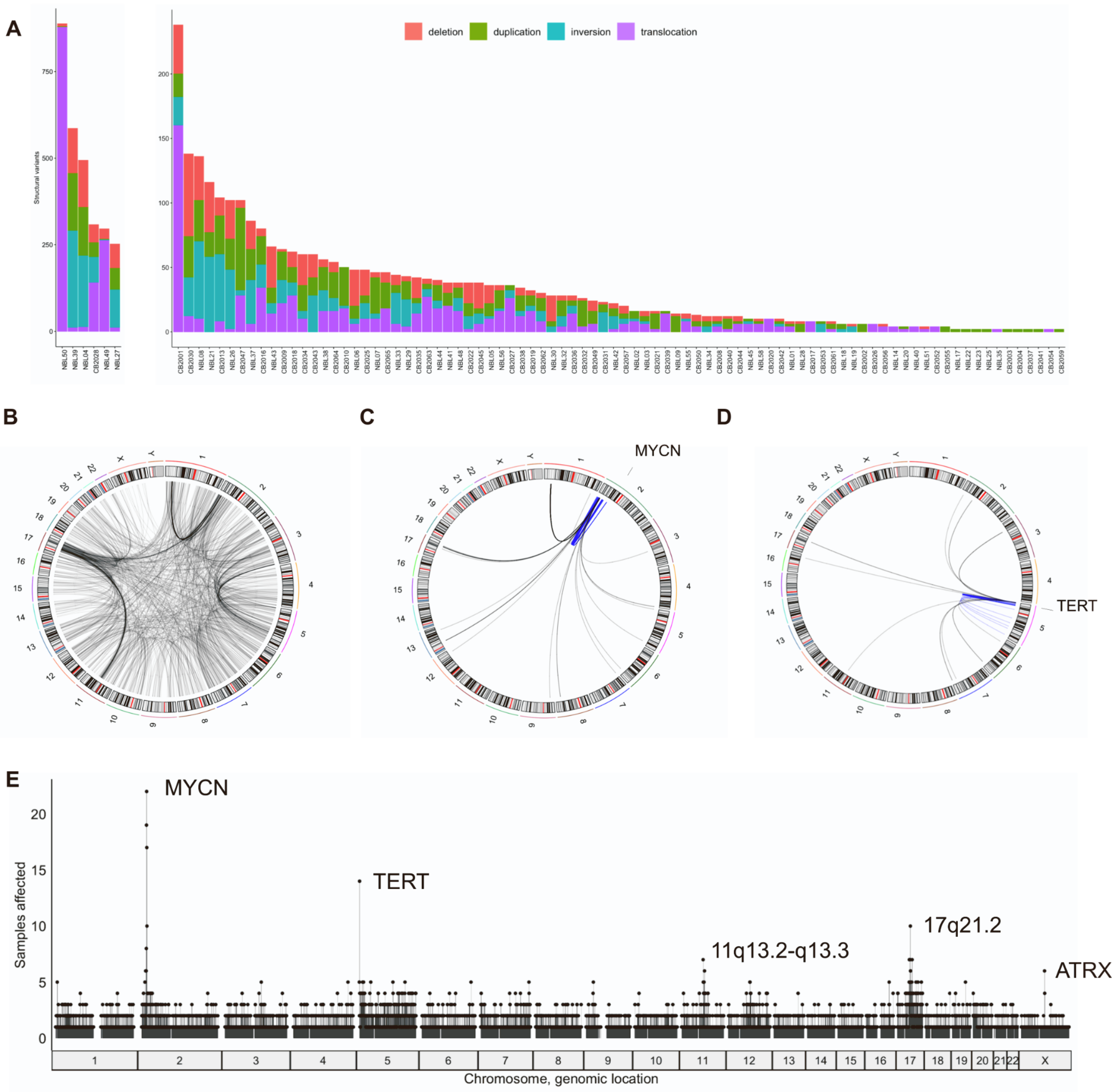

Figure S9. Genome-wide patterns of structural variation, related to Figure 4C

(A) Number of structural variants per class (deletion, duplication, inversion, translocation) and sample. Samples without detected SVs not shown.

(B) Interchromosomal translocations.

[1] https://string-db.org/, network visualization (STRING version 11.0b)

(C) Structural variation with breakpoints in a 5 Mb window around *MYCN.*

(D) Structural variation with breakpoints in a 5 Mb window around *TERT.*

(E) Number of tumors affected by structural variation in 500 kb genomic bins.

Intrachromosomal SVs in (C,D) in blue, others in gray.

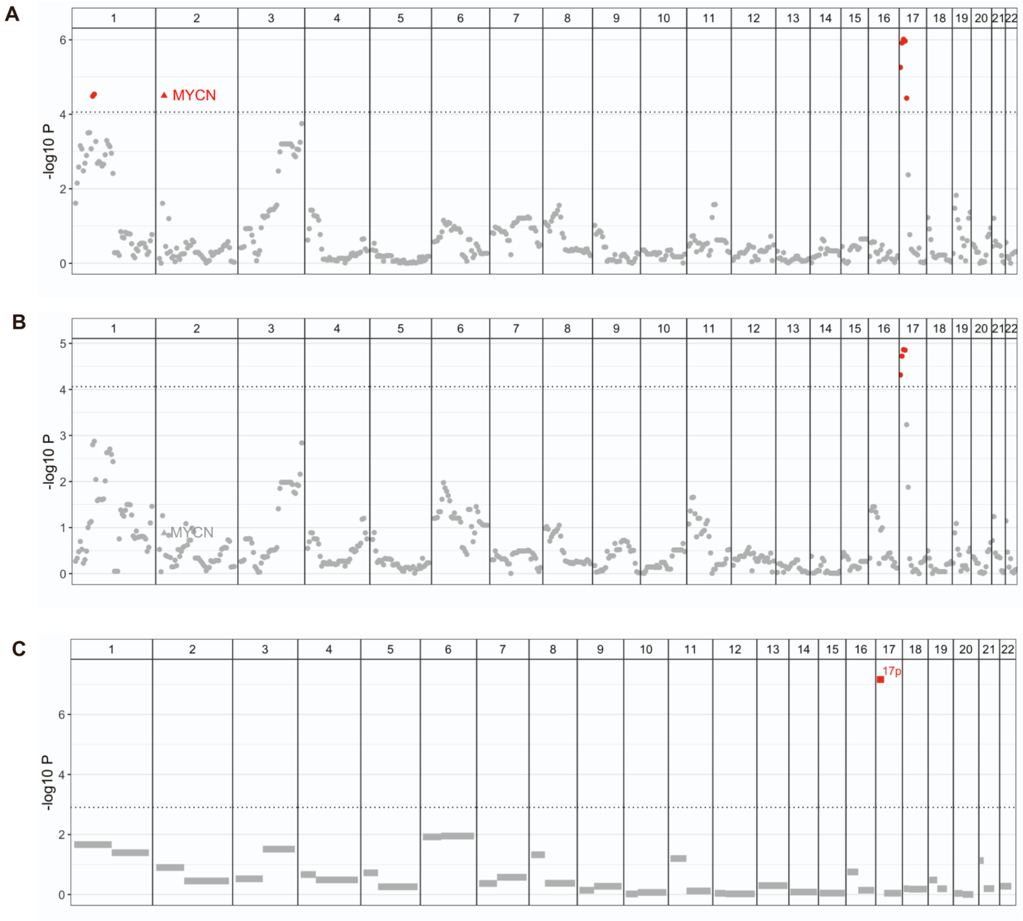

Figure S10. Associations of copy-number ratio and disease-specific survival, related to Figure 2B,C,E and Figure 4A

(A) Association in 5 Mb genomic bins.

(B) Association in 5 Mb bins controlling for *MYCN* amplification status.

(C) Association on the level of chromosome arms. Triangles indicate overlap between the 5 Mb genomic bin and *MYCN*. Copy significant after adjusting p-value for multiple testing in red, others in gray. Significance threshold (FWER 0.05) demarcated by horizontal dotted line.

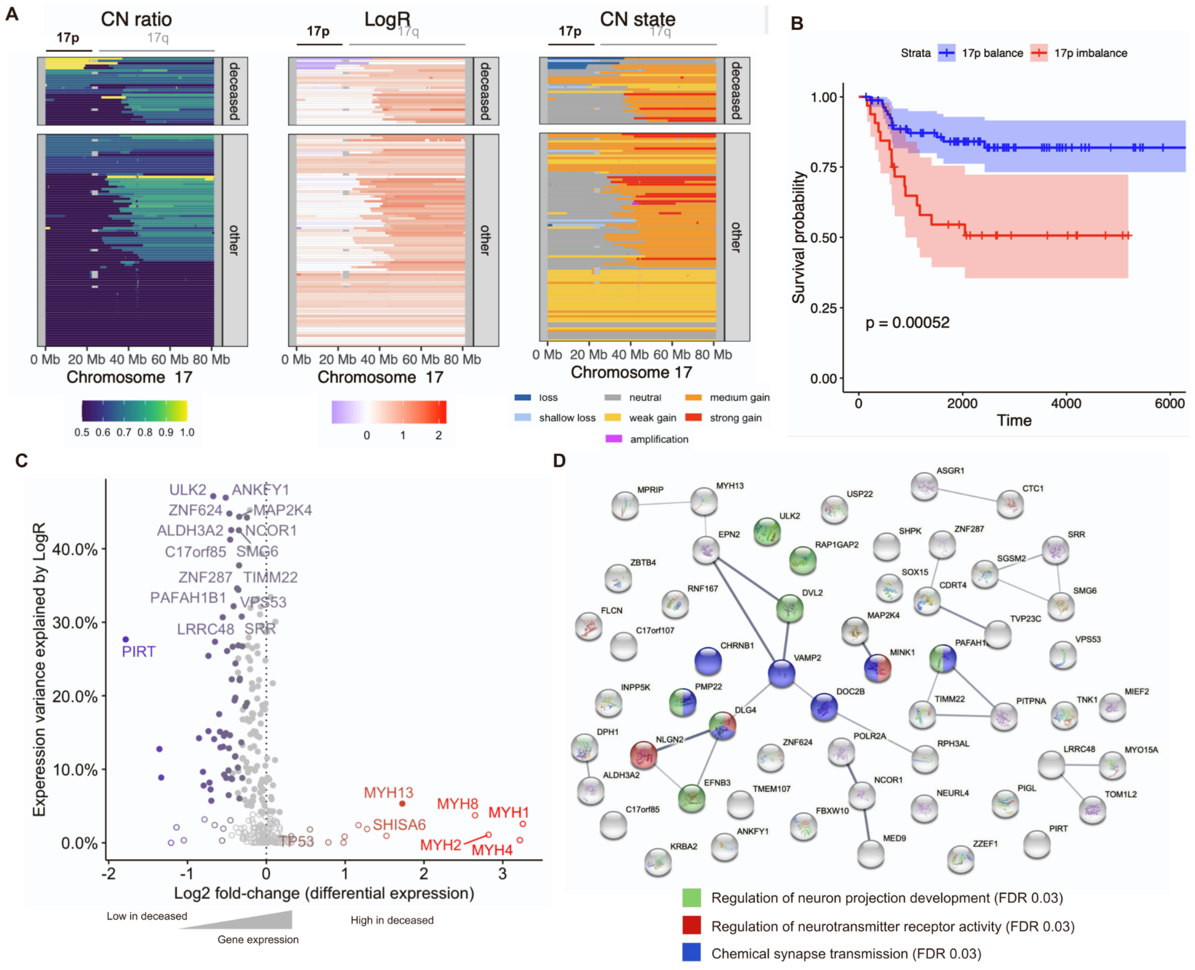

Figure S11. 17p imbalance and copy-number dosage-dependent downregulation of neuronal genes in a subset of unfavorable tumors, related to Figure 2B,C,E

(A) Copy-number ratio, LogR and copy-number state of copy-number segments on chromosome 17 (p and q).

(B) Kaplan-Meier estimate for survival curve by 17p copy-number imbalance. Censored data indicated by vertical marks. Colored ribbons correspond to 95% confidence intervals.

(C) Expression variance explained by LogR and differential expression Log2 fold-change. Differentially expressed genes in color scale, others in light gray. copy-number dosage effect genes as filled circles, others as empty circles. Genes of Log2 fold-change >1.5 or those >30% expression variance explained by LogR labeled by name. *TP53* is additionally labeled.

(D) Protein interaction network of differentially expressed copy-number dosage effect genes on 17p colored by selected biological processes indicated below.

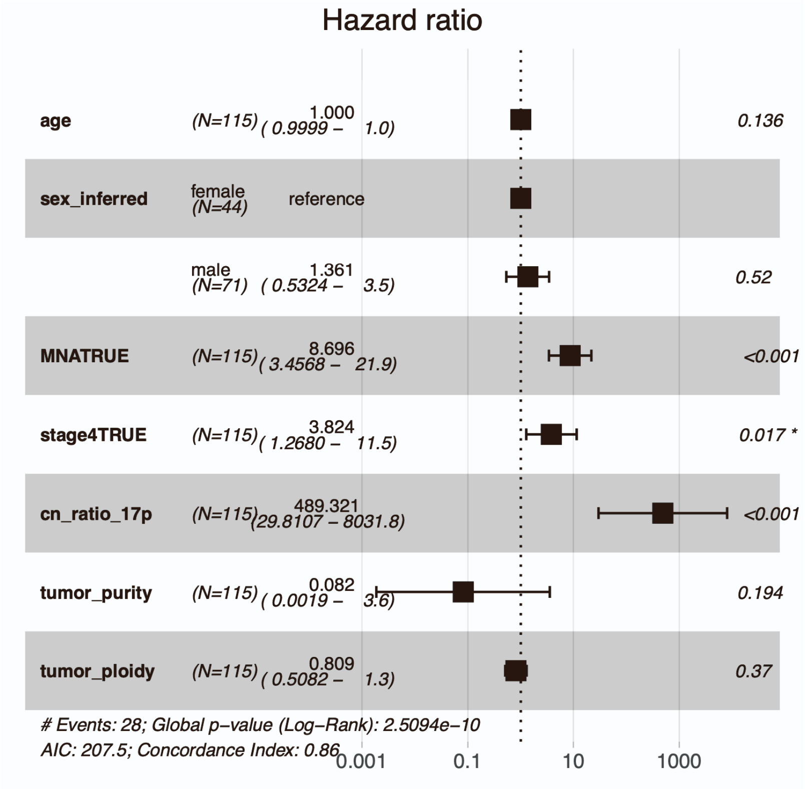

Figure S12. Cox proportional hazards regression model including chromosome 17p imbalance status, related to Figure 2B,C,E and Figure S11

Squares represent point estimates, whiskers indicate 95% confidence interval of estimate.

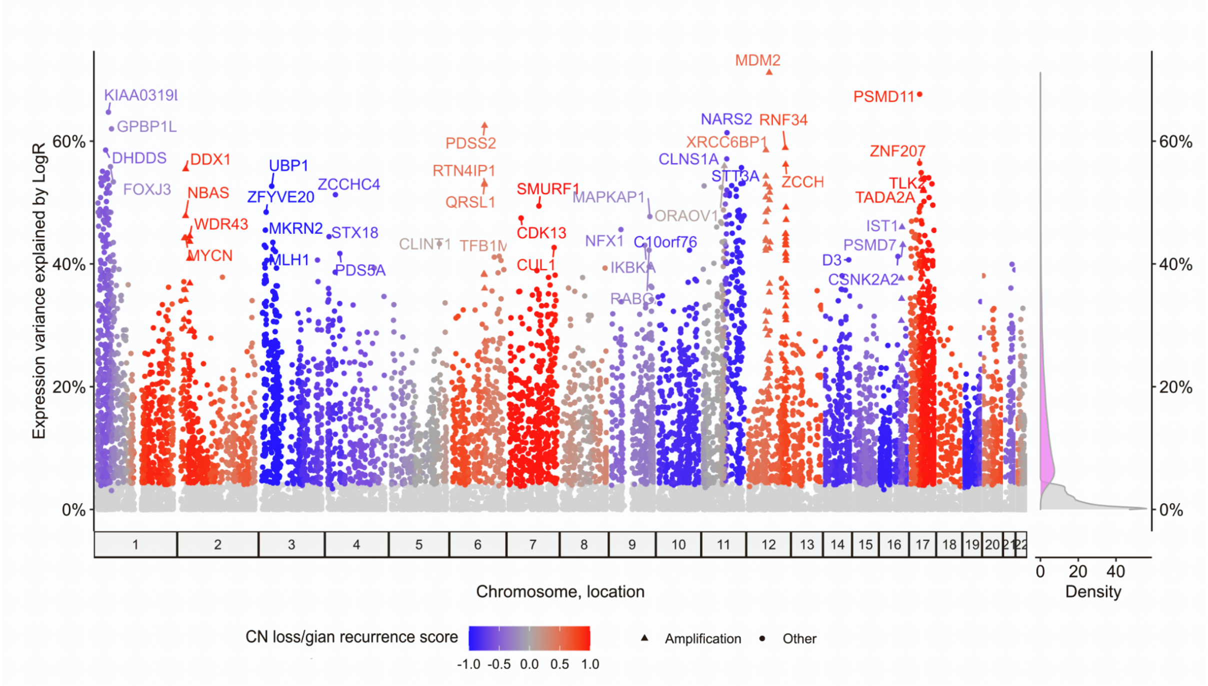

Figure S13. Genome-wide copy-number dosage effects, related to Figure 2A,B,D,E,F

Dosage effect as variance of total expression explained by LogR per gene. Dark gray and shades of red and blue indicate significant dosage copy-number effect genes (FDR < 0.05, Benjamini-Hochberg). Non-significant genes in light gray. Color scale indicates copy-number recurrency score with shades of red and blue depicting recurrent copy-number gains and losses respectively. Genes amplified in one or more tumors as triangles. Top four genes with at least 40% variance explained per chromosome are labeled. Right: Densities of percent variance explained for significant genes (magenta) and non-significant genes (light gray).

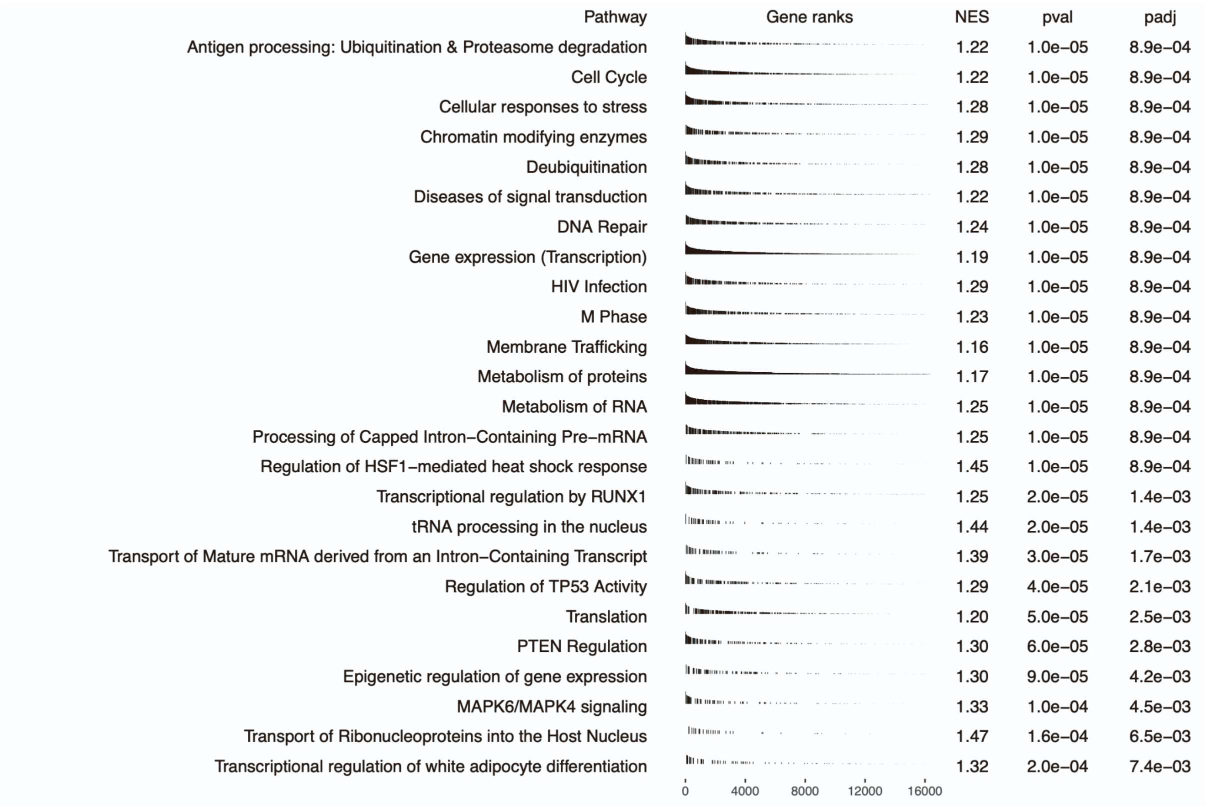

Figure S14. Pathways enriched in copy-number dosage effects on gene expression, related to Figure 2A,B,D,H

Independent reactome pathways enriched for copy-number dosage effect on gene expression. Black bars in column “Gene ranks” indicate pathway membership of genes ranked by copy-number dosage effect. NES, normalized enrichment score.

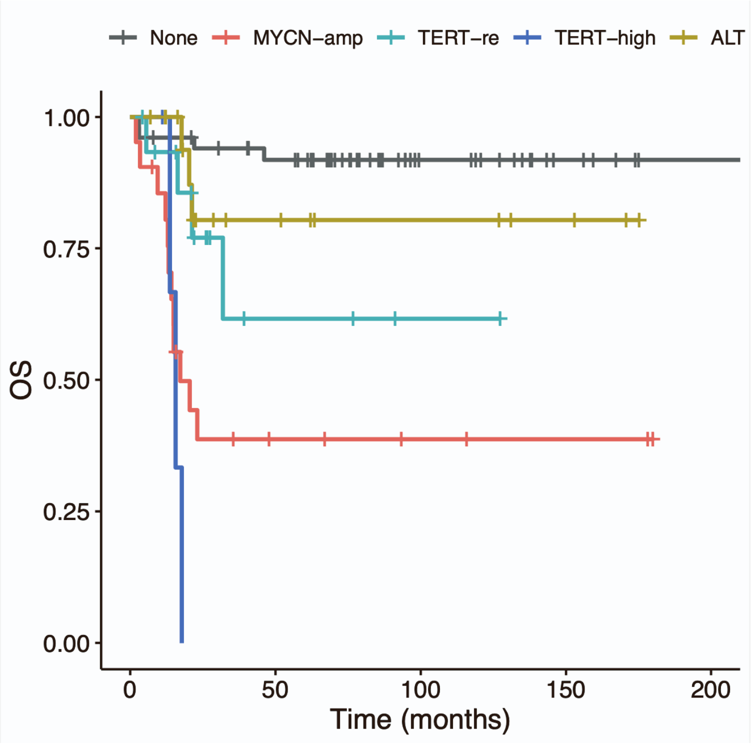

Figure S15. Kaplan-Meier estimate of overall survival (OS) by molecular telomere maintenance features, related to Figure 3C

Molecular telomere maintenance features: *MYCN* amplification (*MYCN*-amp), *TERT* rearrangement (*TERT*-re), high *TERT* expression and lack of *MYCN*-amp and *TERT*-re (*TERT*-high) and alternative lengthening of telomeres (ALT). OS, Overall survival.

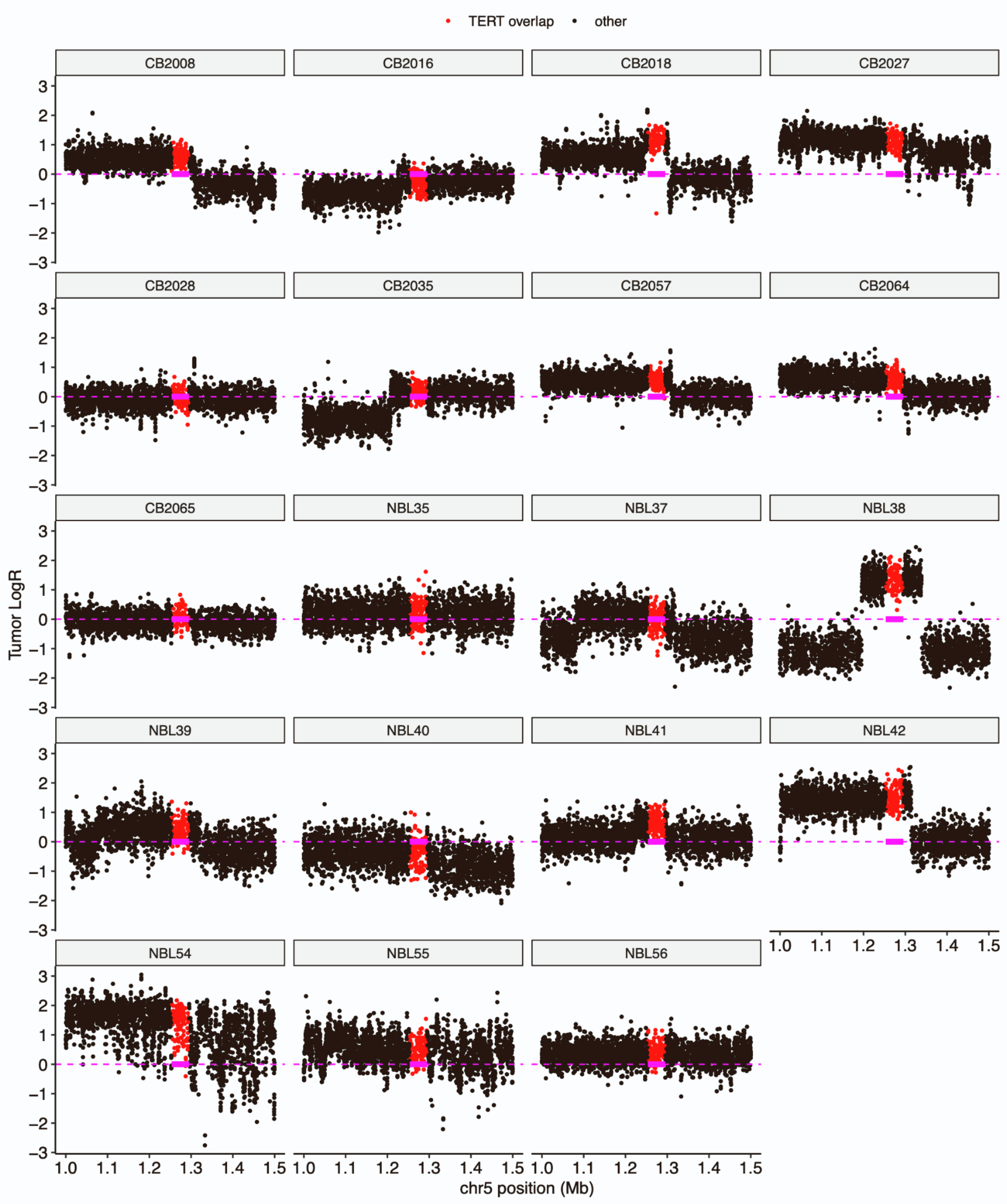

Figure S16. Somatic copy-number alterations affecting the TERT locus in TERT-rearranged tumors, related to Figure 3E

Tumor LogR of *TERT*-rearranged tumors at individual base pair positions (1000 Genomes SNPs) show gains and losses at the broader *TERT* locus on chromosome 5 (1.0 – 1.5 Mb). LogR measurements overlapping the *TERT* gene in red, others in black. Baseline (tumor LogR = 0) indicative of unaltered (normal) copy-number and *TERT* gene boundaries as magenta dashed line and magenta box respectively.

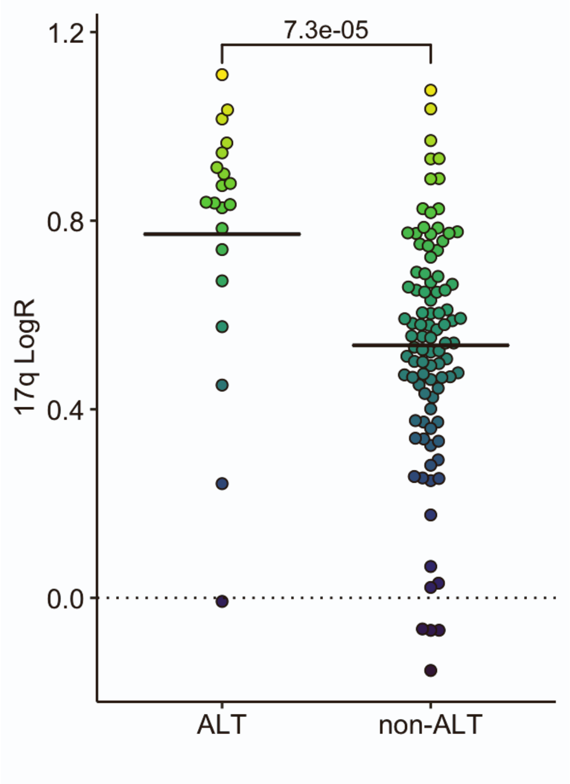

Figure S17. Log-ratio of 17q coverage difference between tumor and normal sample by alternative lengthening of telomeres status, related to Figure 1C and Figure 4

P-value between groups determined by one sided Wilcox rank sum test. ALT, alternative lengthening of telomeres. Horizontal bars indicate group mean.

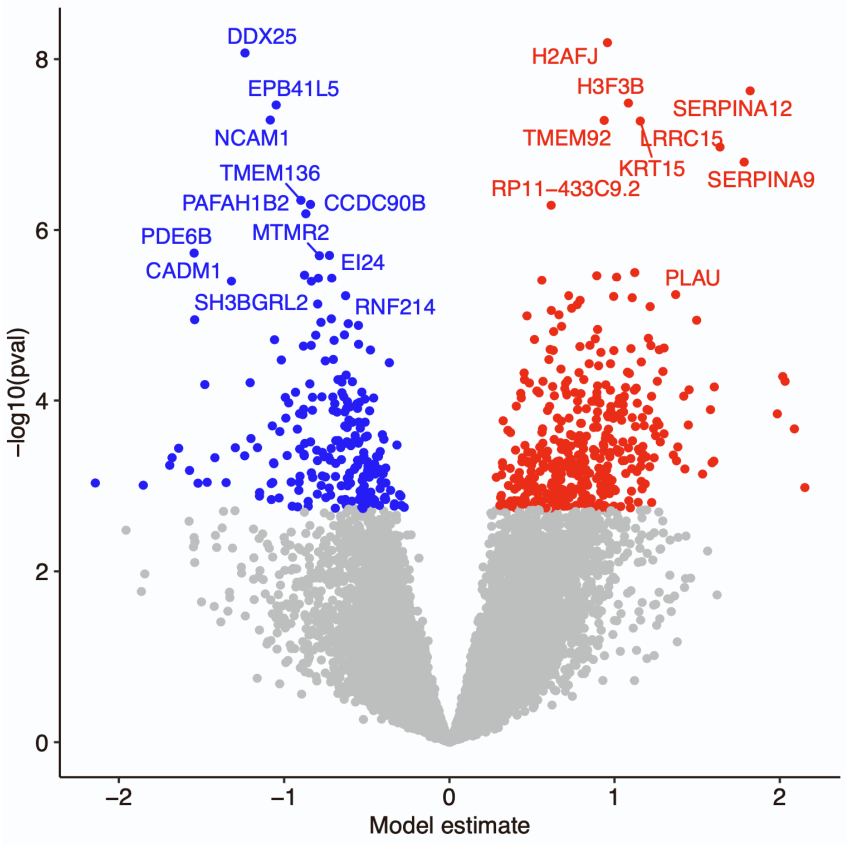

Figure S18. Differential expression analysis between ALT and non-ALT tumors, related to Figure 4D

Red indicates upregulated genes in ALT, blue indicates ALT down-regulated genes (FDR < 0.05). ALT, alternative lengthening of telomeres.

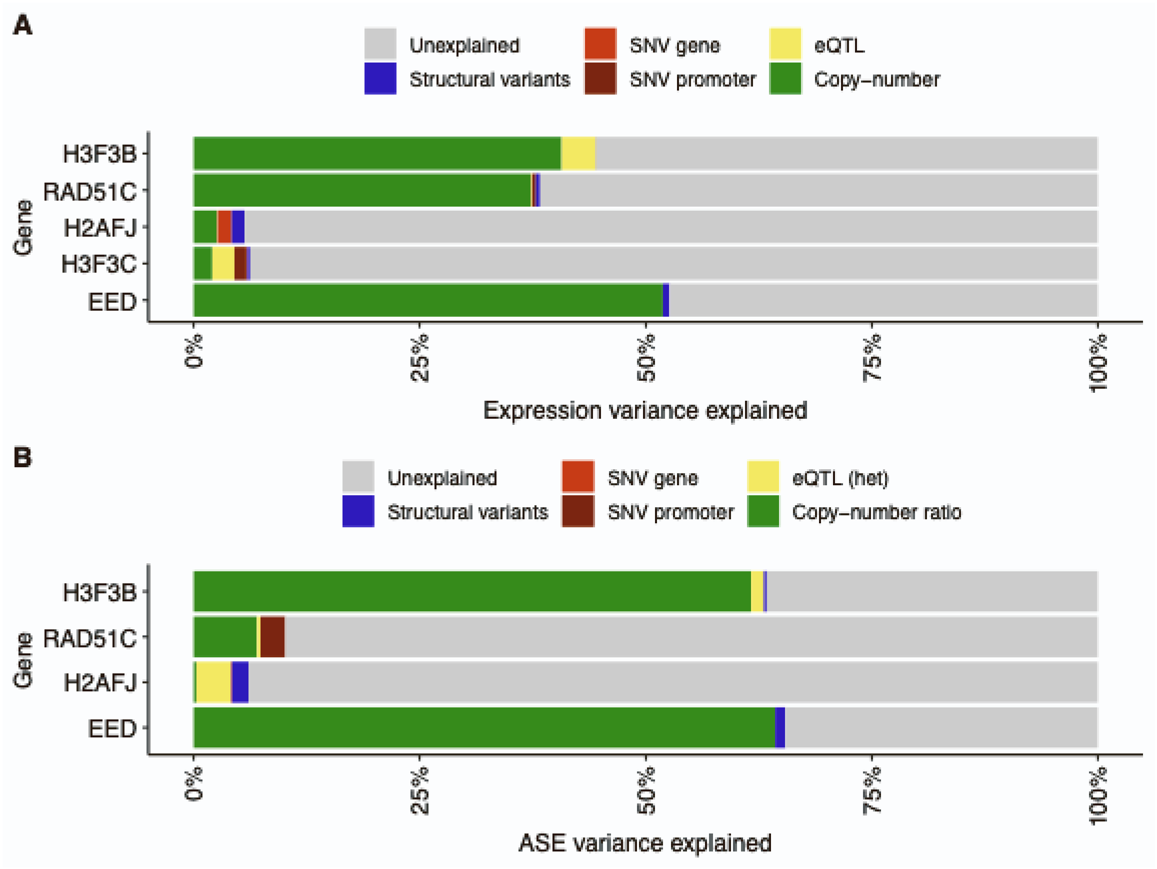

Figure S19. Local genetic effects on expression and ASE in H3F3B, RAD51C, H2AFJ, H3F3C and EED, related to Figure 4D,E

(A) Variance in gene expression explained by genetic effects.

(B) Variance in ASE explained by genetic effects. ASE variance of gene *H3F3C* could not be investigated, because variance components were only determined for genes for which at least 20 tumors harbored at least one heterozygous expressed SNP, which is required to calculate the ASE ratio. However, heterozygous expressed SNPs within *H3F3C* were only found in a single tumor (NBL50).

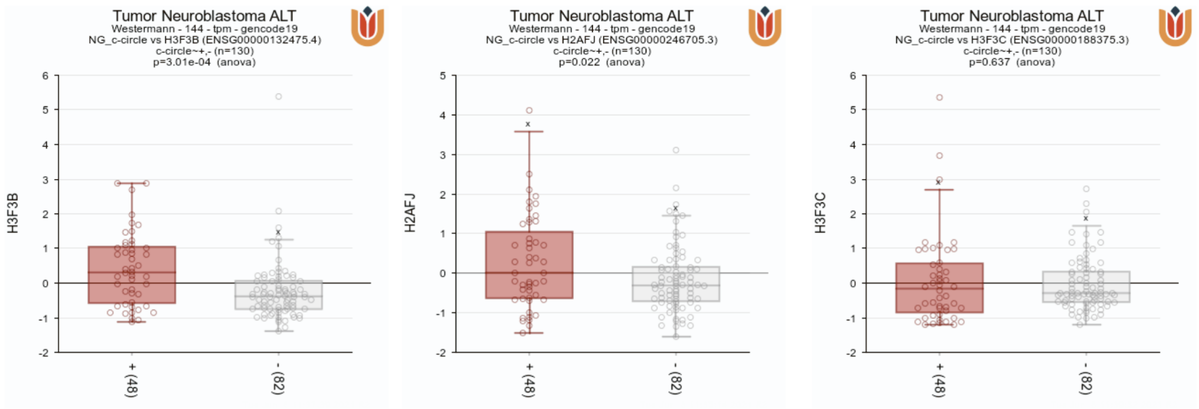

Figure S20. H3F3B, H3F3C and H2AFJ expression in c-circle positive and negative neuroblastoma tumors, related to Figure 4D,E

Expression z-scores of histone variant genes *H3F3B*, *H2AFJ* and *H3F3C* in 130 neuroblastoma tumors stratified by positive (+) (n = 48) and negative (-) (n = 82) c-circle status from Hartlieb et. al 2021 (R2: Genomics Analysis and Visualization Platform, http://r2.amc.nl). Boxplot midlines mark median; upper and lower hinges extend to first and third quartile; upper and lower whiskers extend to the smallest and largest value max. 1.5 × IQR;

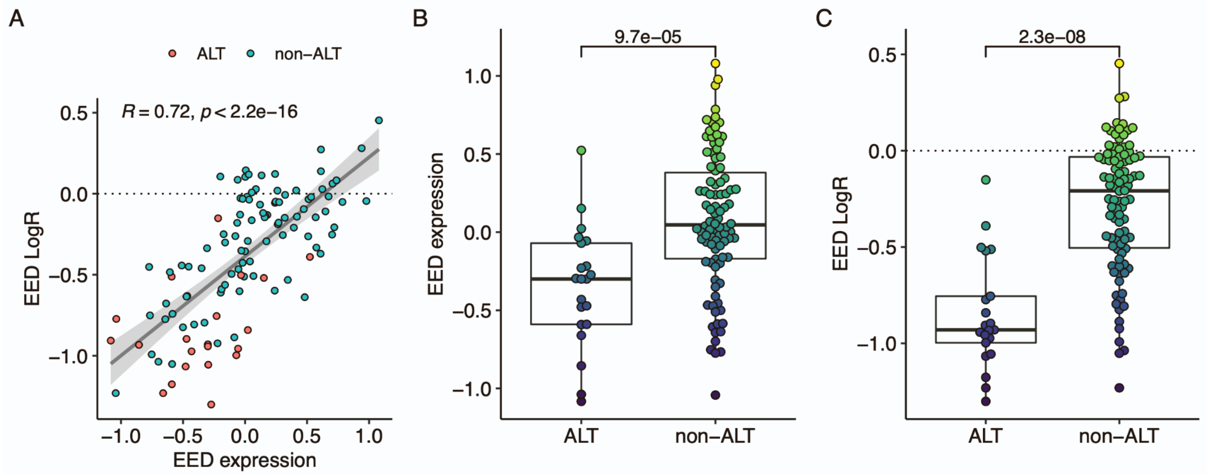

Figure S21. Copy-number Log-ratio (LogR) and expression of the EED in ALT and non-ALT neuroblastomas, related to Figure 4

(A) Linear regression of copy-number LogR and gene expression values for gene *EED* per tumor. Grey ribbon indicates 95% confidence interval of regression line.

(B) *EED* gene expression by ALT status.

(C) *EED* gene copy-number LogR by ALT status.

Boxplot midlines in (B,C) mark median; upper and lower hinges extend to first and third quartile; upper and lower whiskers extend to the smallest and largest value max. 1.5 × IQR; P-value of Wilcox rank sum test between ALT and non-ALT tumors in (B,C) shown above bracket.

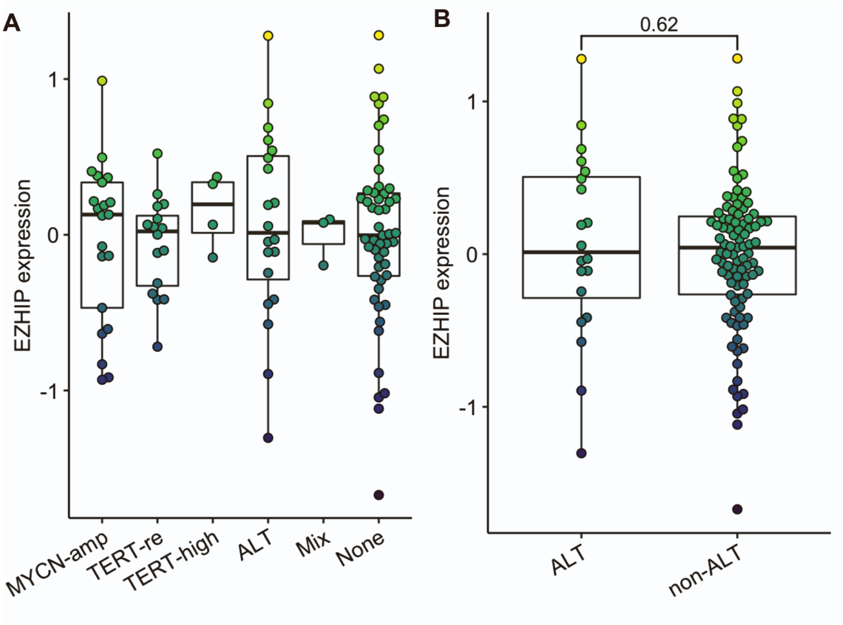

Figure S22. EZHIP expression by molecular telomere maintenance features, related to Figure 4E,F

(A) Normalized gene expression of PRC2 inhibitor *EZHIP* by molecular telomere maintenance group.

(B) Normalized gene expression of *EZHIP* by ALT status; P-value of Wilcox rank sum test between ALT and non-ALT tumors shown above bracket.

Boxplot midlines mark median; upper and lower hinges extend to first and third quartile; upper and lower whiskers extend to the smallest and largest value max. 1.5 × IQR.

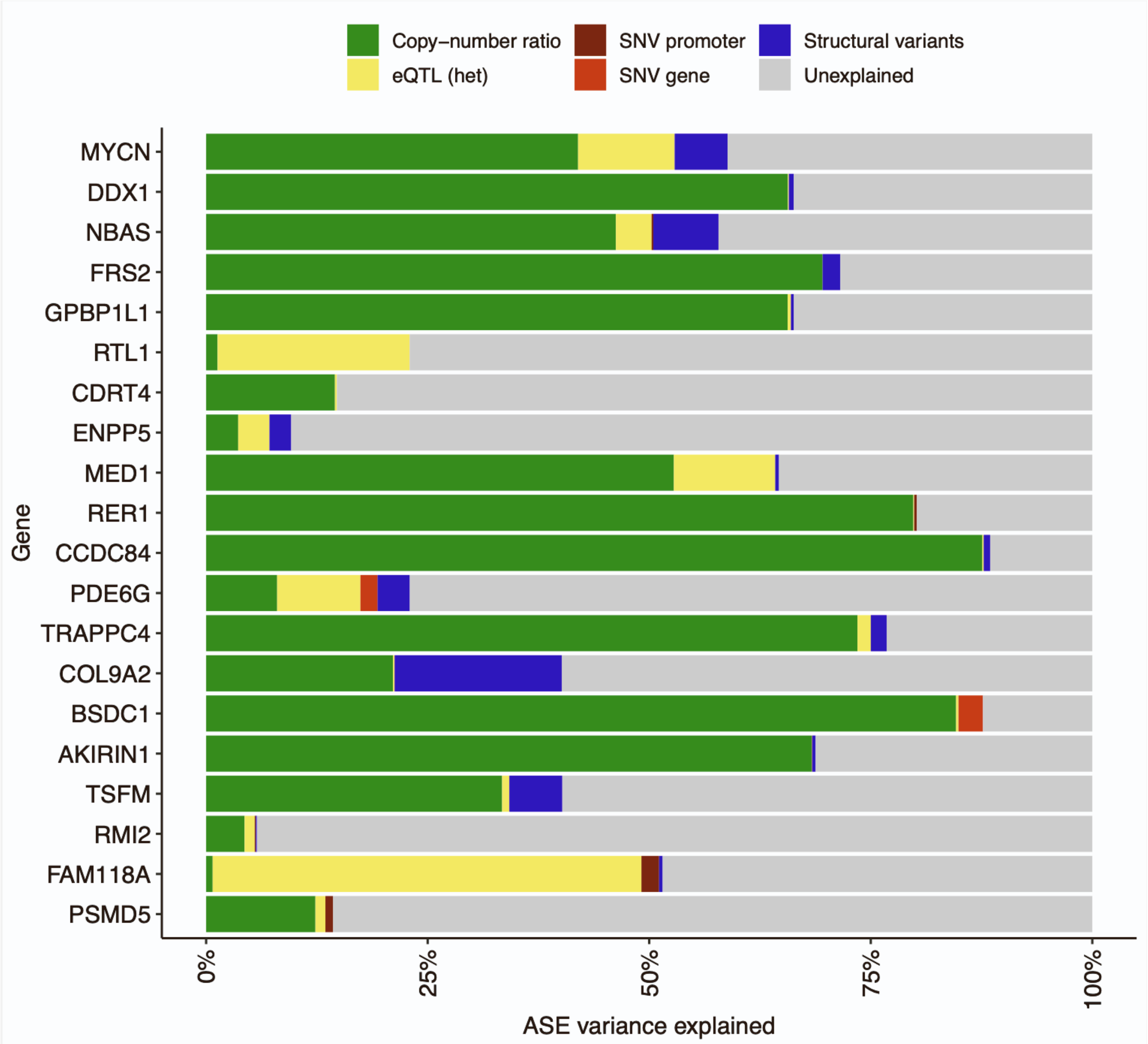

Figure S23. Contribution of local genetic effects on ASE in allelic dosage genes (top 20 genes with strongest ASE–expression correlation), related to Figure 5C

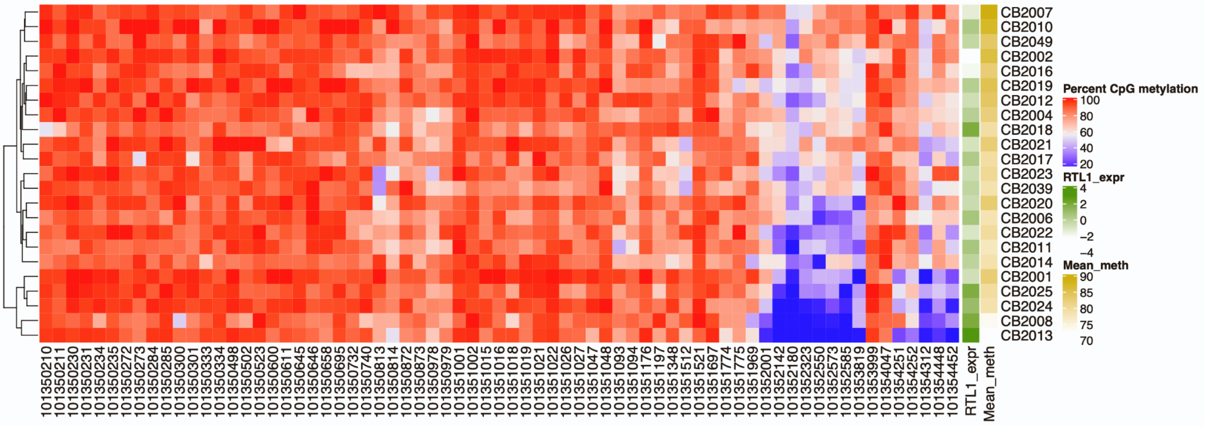

Figure S24. CpG methylation in the RTL1 upstream region, related to Figure 5D-G

Heatmap shows percent methylated CpGs from whole-genome bisulfite sequencing of the *RTL1* upstream region in a -4kb to +1kb window relative to *RTL1* gene start. Chromosome 14 genomic location of CpG denoted on x-axis.

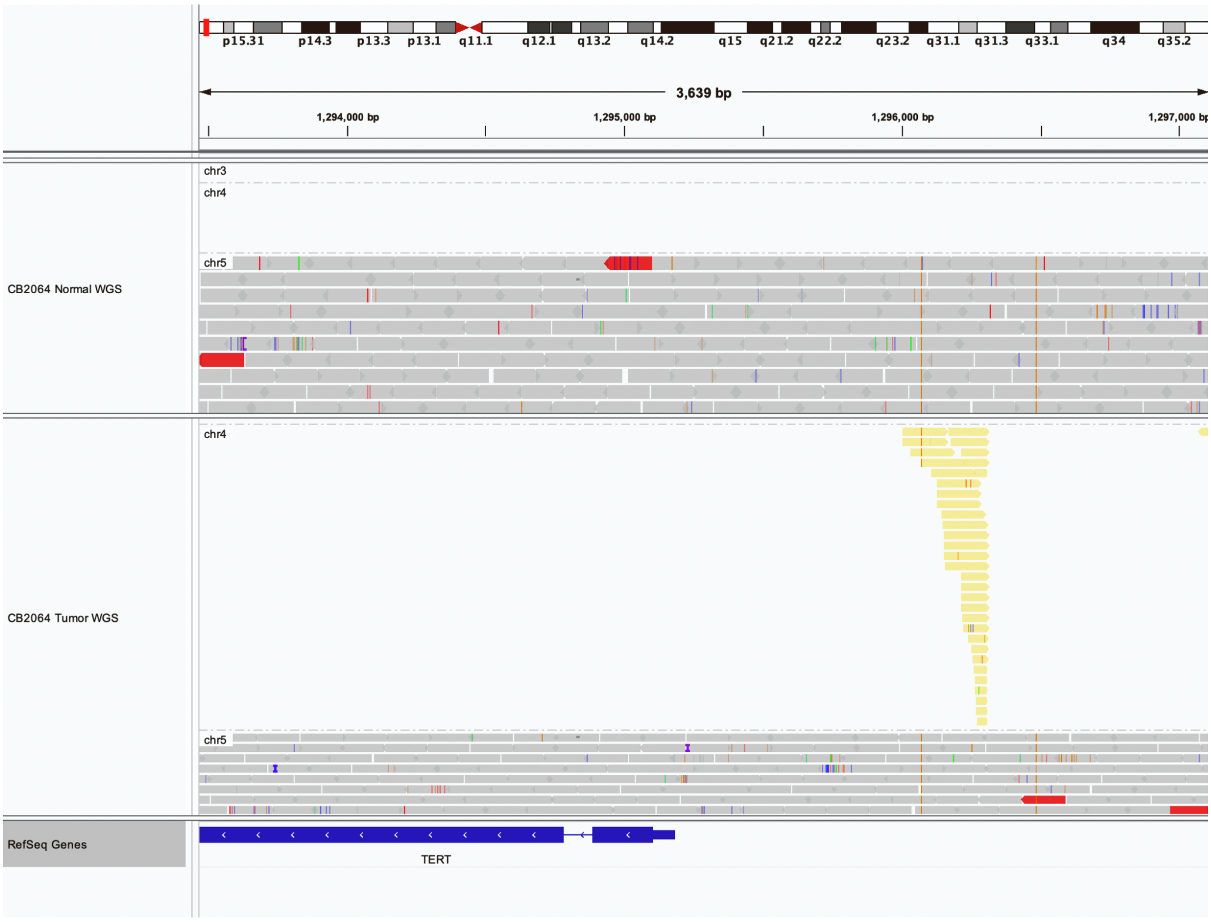

Figure S25. TERT promoter rearrangement in CB2064, related to STAR methods and Figure 1C

Discordant paired-end read mapping between *TERT* promoter and chromosome 4 in tumor CB2064. Aligned reads with paired-end mates mapping to chromosome 4 are indicated in yellow and found exclusively in tumor WGS (bottom) but not in normal WGS (top).

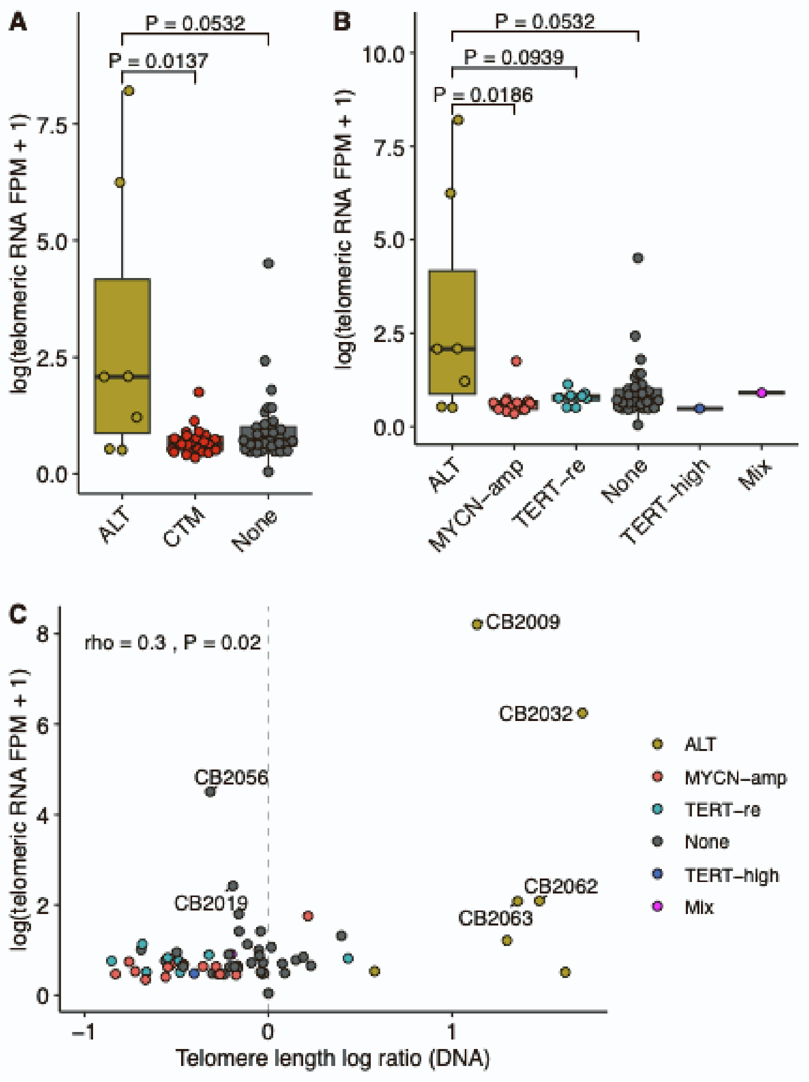

Figure S26. Abundance of telomere RNA in N=63 neuroblastoma tumors, related to STAR methods and Figure 1C

(A) Abundance of telomeric RNA between telomere maintenance categories.

(B) Abundance of telomeric RNA by molecular subtypes.

(C) Abundance of telomeric RNA and relative telomere length between tumor and normal sample as determined from whole-genome sequencing. rho: Spearman's correlation coefficient. FPM, fragments per million.

Boxplot midlines in (A,B) mark median; upper and lower hinges extend to first and third quartile; upper and lower whiskers extend to the smallest and largest value max. 1.5 × IQR; Differences determined by two-sided Wilcox rank sum test. ALT: alternative lengthening of

telomeres; CTM: canonical telomere maintenance; MYCN-amp: MYCN amplification; TERT-re: TERT-rearrangement.

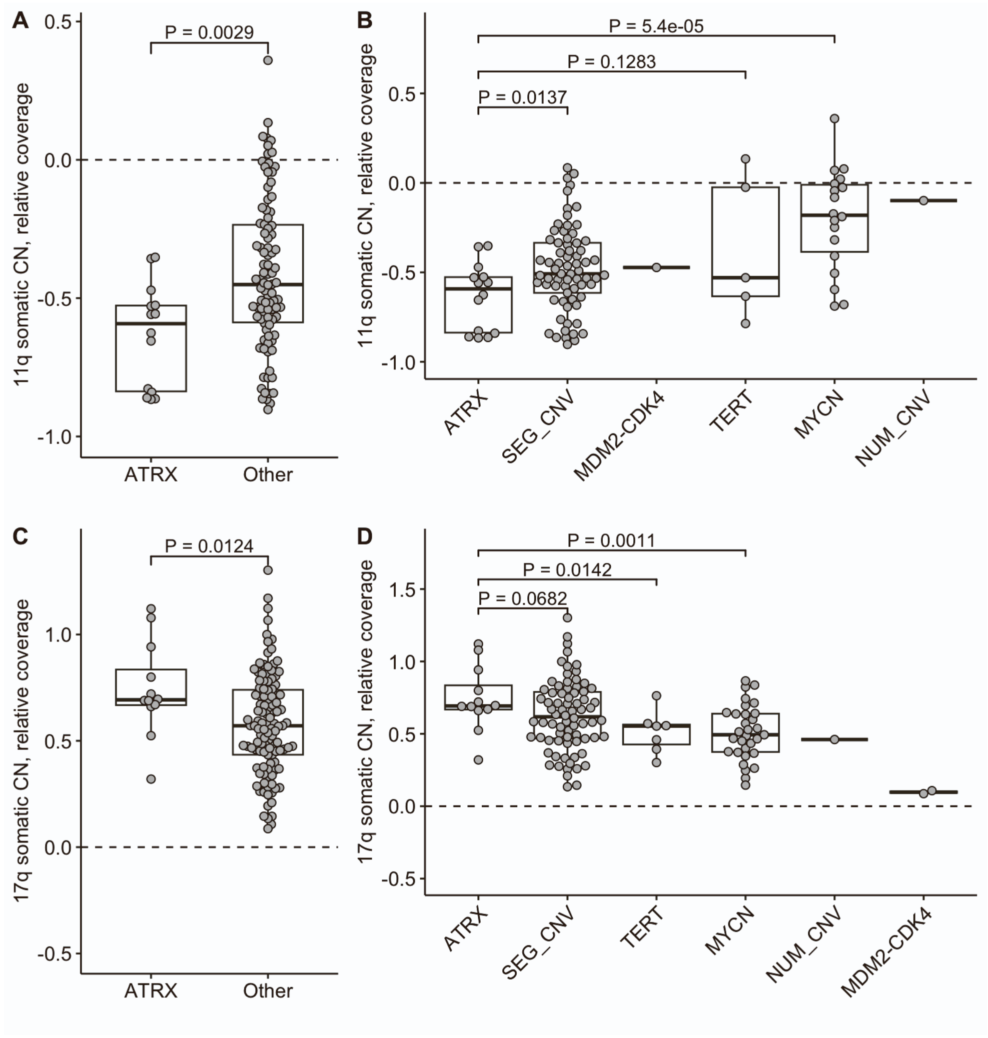

Figure S27. Somatic copy number by relative coverage differences between tumor and normal samples reported by Gundem et al. 2023 [3], related to Figure 4A-D

(A) 11q copy-number in ATRX-mutated and all other tumors.

(B) Somatic 11q copy-number differences between ATRX-mutated and other neuroblastoma molecular subtypes reported by Gundem and colleagues.

(**C)** Somatic 17q copy-number differences between ATRX and all other tumors.

(**D)** Somatic 17q copy-number differences between ATRX and other molecular subtypes reported by Gundem and colleagues.

Boxplot midlines mark median; upper and lower hinges extend to first and third quartile; upper and lower whiskers extend to the smallest and largest value max. 1.5 × IQR. Differences determined by two-sided Wilcox rank sum test.

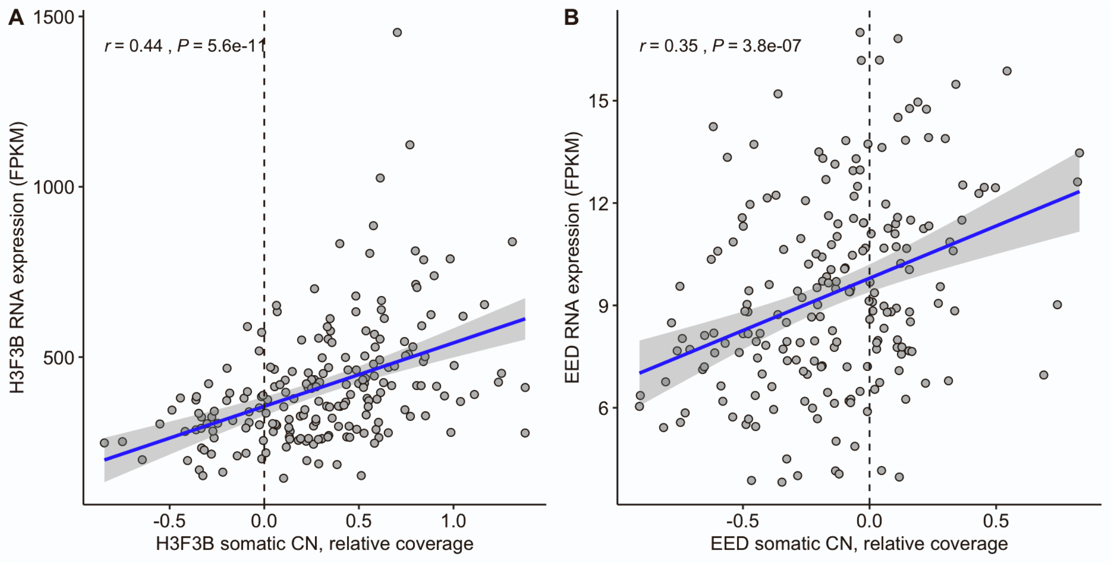

Figure S28. Copy-number dosage effects on expression of H3F3B and EED genes from Egolf et al 2019, related to Figure 4D-E

(A) Copy-number dosage effects on gene expression of H3F3B.

(B) Copy-number dosage effects on gene expression of EED.

RNA-seq and somatic copy-number data deposited for neuroblastoma study phs001436 [4]. Regression line shown in blue, 95% confidence interval in gray. Test for Pearson's correlation coefficient (r); FPKM, fragments per kilobase per million; CN, copy-number.

References

1. Ackermann, S., Cartolano, M., Hero, B., Welte, A., Kahlert, Y., Roderwieser, A., Bartenhagen, C., Walter, E., Gecht, J., Kerschke, L., et al. (2018). A mechanistic classification of clinical phenotypes in neuroblastoma. Science *362*, 1165–1170.

2. Hartlieb, S.A., Sieverling, L., Nadler-Holly, M., Ziehm, M., Toprak, U.H., Herrmann, C., Ishaque, N., Okonechnikov, K., Gartlgruber, M., Park, Y.-G., et al. (2021). Alternative lengthening of telomeres in childhood neuroblastoma from genome to proteome. Nat. Commun. *12*, 1269.

3. Gundem, G., Levine, M.F., Roberts, S.S., Cheung, I.Y., Medina-Martínez, J.S., Feng, Y., Arango-Ossa, J.E., Chadoutaud, L., Rita, M., Asimomitis, G., et al. (2023). Clonal evolution during metastatic spread in high-risk neuroblastoma. Nat. Genet. *55*, 1022–1033.

4. Egolf, L.E., Vaksman, Z., Lopez, G., Rokita, J.L., Modi, A., Basta, P.V., Hakonarson, H., Olshan, A.F., and Diskin, S.J. (2019). Germline 16p11.2 Microdeletion Predisposes to Neuroblastoma. Am. J. Hum. Genet. *105*, 658–668.
